# Thermodynamically consistent Bayesian analysis of closed biochemical reaction systems

**DOI:** 10.1186/1471-2105-11-547

**Published:** 2010-11-05

**Authors:** Garrett Jenkinson, Xiaogang Zhong, John Goutsias

**Affiliations:** 1 Whitaker Biomedical Engineering Institute, The Johns Hopkins University, Baltimore, MD 21218, USA; 2 Department of Applied Mathematics and Statistics, The Johns Hopkins University, Baltimore, MD 21218, USA

## Abstract

**Background:**

Estimating the rate constants of a biochemical reaction system with known stoichiometry from noisy time series measurements of molecular concentrations is an important step for building predictive models of cellular function. Inference techniques currently available in the literature may produce rate constant values that defy necessary constraints imposed by the fundamental laws of thermodynamics. As a result, these techniques may lead to biochemical reaction systems whose concentration dynamics could not possibly occur in nature. Therefore, development of a thermodynamically consistent approach for estimating the rate constants of a biochemical reaction system is highly desirable.

**Results:**

We introduce a Bayesian analysis approach for computing thermodynamically consistent estimates of the rate constants of a closed biochemical reaction system with known stoichiometry given experimental data. Our method employs an appropriately designed prior probability density function that effectively integrates fundamental biophysical and thermodynamic knowledge into the inference problem. Moreover, it takes into account experimental strategies for collecting informative observations of molecular concentrations through perturbations. The proposed method employs a maximization-expectation-maximization algorithm that provides thermodynamically feasible estimates of the rate constant values and computes appropriate measures of estimation accuracy. We demonstrate various aspects of the proposed method on synthetic data obtained by simulating a subset of a well-known model of the EGF/ERK signaling pathway, and examine its robustness under conditions that violate key assumptions. Software, coded in MATLAB^®^, which implements all Bayesian analysis techniques discussed in this paper, is available free of charge at http://www.cis.jhu.edu/~goutsias/CSS%20lab/software.html.

**Conclusions:**

Our approach provides an attractive statistical methodology for estimating thermodynamically feasible values for the rate constants of a biochemical reaction system from noisy time series observations of molecular concentrations obtained through perturbations. The proposed technique is theoretically sound and computationally feasible, but restricted to quantitative data obtained from closed biochemical reaction systems. This necessitates development of similar techniques for estimating the rate constants of open biochemical reaction systems, which are more realistic models of cellular function.

## Background

Biochemical reaction systems are popular models of cellular function. These models are extensively used to represent the inter-connectivity and functional relationships among molecular species in cells and, most often, they provide accurate description of cellular behavior. Inferring a biochemical reaction system from experimental data is an important step towards building mathematical and computational tools for the analysis of cellular systems. This step requires both structure (stoichiometry) identification as well as parameter (rate constant) estimation [1-4]. Due however to the large combinatorial complexity of determining the stoichiometry of a biochemical reaction system, solving this problem requires large amounts of high quality experimental data and substantial computational resources, which are not usually available in practice.

Recently, several approaches have been proposed in the literature for addressing a simpler problem, known as *model calibration*. The objective of model calibration is to adjust the kinetic parameters of a biochemical reaction system with given stoichiometry in order to obtain a sufficiently good match between simulated and observed dynamics; e.g. see [2,5-11].

Among known model calibration techniques, the ones based on Bayesian analysis [7,10,11] are perhaps the most versatile. Bayesian analysis allows us to effectively incorporate biophysical knowledge into the problem at hand and naturally draw statistical conclusions about the unknown kinetic parameters. This is done by employing a probability density function that encapsulates prior information about the rate constants of a biochemical reaction system and by deriving a posterior probability density function over the kinetic parameters after experimental data have been collected. By taking into account the experimental data and the information contained in the prior, the posterior density summarizes all knowledge available about the unknown kinetic parameters and quantifies uncertainty about their true values [12,13]. Moreover, the posterior allows us to quantify our confidence about estimation accuracy, compute probabilities over alternative calibrations, and design additional experiments to improve inference.

Most published model calibration techniques do not take into account constraints on the reaction rate constants imposed by the fundamental laws of thermodynamics. If these constraints, known as Wegscheider conditions [14,15], are not explicitly considered by a model calibration technique, then the method will spend most time examining impossible kinetic parameter sets and will most probably produce a biochemical reaction system that is not physically realistic [16]. This issue has been recently recognized in the literature, and new modeling formalisms have been suggested in an effort to address it [17-20]. The proposed formalisms describe a biochemical reaction system by well-defined thermodynamic parameters whose values always guarantee that the reaction rate constants satisfy the Wegscheider conditions. For example, in [19,20], a biochemical reaction system is parameterized in terms of molecular capacities and reaction resistances, by using a thermodynamic kinetic modeling (TKM) formalism that enjoys a number of advantages over the ones suggested in [17,18].

We believe that parameterizing a biochemical reaction system in terms of capacities and resistances is unnecessary and, in certain instances, problematic. It has been pointed out in [19] that different choices for the TKM parameters can lead to the same concentration dynamics. As a consequence, the TKM parameters cannot be determined uniquely from concentration measurements. A way to address this problem is to take the capacities to be the equilibrium concentrations (which is always possible in closed biochemical reaction systems), in which case the capacities are constrained by conservation relationships imposed by the system stoichiometry. Then, parameter estimation in the TKM formalism may be possible by arbitrarily fixing a subset of capacity values and estimating the remaining capacities and resistances. However, this approach can be very cumbersome when dealing with molecular perturbations (as we do in this paper) or when merging estimated TKM models, since, in both cases, the capacities may not refer to compatible equilibrium concentrations. It has been suggested in [19] that a way to merge two models using the TKM formalism is to first convert the capacities and resistance to the rate parameters, merge the two models, and then convert back to the TKM formalism. However, this approach seems to be overly complicated, especially in view of the model calibration methodology presented here.

In this paper, we introduce a thermodynamically consistent Bayesian analysis approach to model calibration that does not require reparametrization. Our approach relies on statistically modeling the reaction rate constants of the forward reactions as well as the equilibrium constants of individual reactions. We restrict our attention to closed systems (or systems that can be approximately considered to be closed), since thermodynamic analysis of such systems is easier to handle than open systems. The proposed approach controls thermodynamic consistency of the reaction rate constants by employing well-defined relationships between the kinetic parameters of a biochemical reaction system, imposed by the Wegscheider conditions. By embedding these relationships within an iterative algorithm that finds the mode of the posterior density, we arrive at a thermodynamically consistent Bayesian estimate for the rate constants.

Bayesian analysis can be appreciably influenced by the choice of the prior probability density functions. This is particularly true in systems biology problems in which only a small number of observations is usually available. It is therefore important to focus our effort on constructing appropriate prior densities for the unknown rate constants of the forward reactions and the equilibrium constants of individual reactions. Although a number of choices may be possible, it is imperative to use fundamental biophysical and thermodynamic principles to derive informative prior densities that effectively encapsulate such principles.

By using the classical Arrhenius formula of chemical kinetics [21], we construct an appropriate prior density for the log-rate constants of the forward reactions. To do so, we assume that the prefactor and activation energy associated with the Arrhenius formula are both random variables following log-normal and exponential distributions, respectively. This approach takes into account unpredictable changes in biochemical conditions affecting the structure of the reactant molecules and the probability of reaction after collision. On the other hand, by exploiting the thermodynamic relationship between rate constants, equilibrium concentrations, and stoichiometric coefficients, we derive an analytical expression for the joint prior density of the logarithms of the equilibrium constants. This expression depends on steady-state concentration measurements and on the stoichiometry of the biochemical reaction system under consideration.

Another important issue associated with the inference problem considered in this paper is the need to collect an informative set of measurements that can lead to sufficiently accurate parameter estimation. It has been increasingly recognized in the literature that a powerful approach to accomplish this goal in problems of systems biology is to selectively perturb key molecular components and measure the effects of these perturbations on the underlying concentrations [22-24]. We follow this strategy here and assume that we can selectively perturb, one at a time, the initial concentrations of a selected number of molecular species in a biochemical reaction system, by increasing or decreasing their values without altering the underlying stoichiometry. This can be achieved by a variety of experimental techniques, such as RNA interference (RNAi), transfection, or molecular injection. Therefore, our approach combines Bayesian analysis with current experimental practices, thus bridging the gap between statistical inference approaches and experimental design.

The Bayesian analysis technique discussed in this paper requires numerical evaluation of a number of statistical summaries of the posterior density. Although several methods are available to deal with this problem (e.g., see [25,26]), we employ here a maximization-expectation-maximization (MEM) strategy that calculates a thermodynamically consistent estimate of the reaction rate constants as well as Monte Carlo estimates of posterior summaries used to evaluate the quality of inference. This strategy is based on sequentially combining a powerful stochastic optimization technique, known as simultaneous perturbation stochastic approximation (SPSA) [27], with Markov chain Monte Carlo (MCMC) sampling [25]. Our experience with extensive synthetic experiments, based on data obtained by simulating a subset of a well-known model of the EGF/ERK signaling pathway, indicates that the proposed algorithm is robust, producing excellent estimation results even in cases of high measurement errors and limited time measurements.

This paper is structured as follows. In the "Methods" section, we provide a brief overview of biochemical reaction systems, discuss how to model perturbations, and present a standard statistical model for the measurements. We then outline our Bayesian analysis approach to model calibration and present our choices for the prior and posterior densities. By emphasizing the fact that the prior density must assign zero probability over the thermodynamically infeasible region of the parameter space, and by employing an encompassing prior approach to Bayesian analysis, we derive an appropriate posterior density that satisfies this condition. We finally outline our proposed methodology for computing thermodynamically consistent Bayesian estimates of the kinetic parameters and for assessing estimation accuracy.

In the "Results/Discussion" section, we provide simulation results, based on a subset of a well-established model of the EGF/ERK signal transduction pathway. These results illustrate key aspects of the proposed model calibration methodology and show its potential for producing sufficiently accurate thermodynamically consistent estimates of a biochemical reaction system from noisy time-series measurements of molecular concentrations.

Finally, in the "Conclusions" section, we discuss a key statistical advantage of the proposed model calibration methodology, viewed from a bias-variance tradeoff perspective. Moreover, we provide suggestions for further research to address a number of practical issues associated with model calibration, such as estimating the initial concentrations and their perturbations, dealing with partially observed or missing data, and extending the proposed technique to the case of open biochemical reaction systems.

Extensive mathematical and computational details are required to rigorously formulate, derive, and understand various aspects of the proposed approach. We provide these details in three Additional files accompanying this paper. In Additional file 1, we present a detailed exposition of the underlying theory, whereas, in Additional file 2, we carefully discuss computational implementation. Finally, in Additional file 3, we provide all necessary details pertaining the biochemical reaction system we use in our simulations. Well-documented software, coded in MATLAB^®^, which implements all Bayesian analysis techniques discussed in this paper, is available to interested readers free of charge at http://www.cis.jhu.edu/~goutsias/CSS%20lab/software.html.

## Methods

### Biochemical reaction systems

In this paper, we consider a biochemical reaction system comprised of *N *molecular species *X*_1_, *X*_2_, ..., *X*_*N *_that interact through *M *coupled reactions of the form:

(1) $$\sum_{n=1}^{N}\nu_{nm}X_{n}\overset{k_{2m-1}}{\underset{k_{2m}}{⇄}}\sum_{n=1}^{N}{\nu^{\prime }}_{nm}X_{n}\text{,}\;m\in ℳ:=\{1,2,\ldots ,M\}.$$

The parameters *k*_2*m*-1 _and *k*_2*m *_are the rate constants of the forward and reverse reactions, whereas, *ν*_*nm*_, ${\nu^{\prime }}_{nm}\geq 0$ are the stoichiometries of the reactants and products. Note that *k*_2*m*-1_, *k*_2*m *_> 0, for all *m *∈ ℳ, since irreversible reactions are thermodynamically not possible in a closed biochemical reaction system [19]. We will assume that the system is well-mixed (homogeneous) with constant temperature and volume. We will also assume that the molecular concentrations evolve continuously as a function of time and that all reactions can be sufficiently characterized by the mass action rate law. In this case, we can describe the dynamic evolution of the molecular concentrations in the system by the following chemical kinetic equations:

(2) $$\frac{dx_{n}^{\left(p\right)}(t)}{dt}=\sum_{{}_{m\in ℳ}}s_{nm}\rho_{m}^{\left(p\right)}(t),\;t\in T,n\in N,p\in P,$$

initialized by

(3) $$x_{n}^{\left(p\right)}(0)=\{\begin{array}{ll} c_{p}+\pi_{p}, & \text{if}\;n=p\neq 0\\ c_{n}, & \text{if}\;p=0\text{ or }n\neq p\neq 0, \end{array}$$

where $\rho_{m}^{\left(p\right)}(t)$ is the net flux of the *m*^th ^reaction at time *t*, given by

(4) $$\rho_{m}^{\left(p\right)}(t)=k_{2m-1}\prod_{{}_{i\in N}}{\left[x_{i}^{\left(p\right)}(t)\right]}^{\nu_{im}}-k_{2m}\prod_{{}_{i\in N}}{\left[x_{i}^{\left(p\right)}(t)\right]}^{{\nu^{\prime }}_{im}},$$

*s_nm _*is the *net *stoichiometry coefficient of the *n*^th ^molecular species associated with the *m*^th ^reaction, defined by $s_{nm}:={v^{\prime }}_{nm}-v_{nm}$, and T := [0, *t*_max_] is an observation time window of interest.

Equations (2)-(4) are based on the assumption that we can selectively perturb, one at a time, the concentrations of molecular species in a set P, by increasing or decreasing their values at time *t *= 0 without altering the underlying stoichiometry. For notational convenience, we include 0 in P and assign *p *= 0 to the original unperturbed system. In this case, $x_{n}^{\left(0\right)}(t)$ is the concentration of the *n*^th ^molecular species in the unperturbed system at time *t*, whereas, $x_{n}^{\left(p\right)}(t)$, for *p *≠ 0, is the concentration of the *n*^th ^molecular species at time *t*, obtained by perturbing the initial concentration of the *p*^th ^species. In (3), *π_p _≥ -c_p _*quantifies the perturbation applied on the initial concentration *c_p _*of the *p*^th ^molecular species at time *t *= 0. When *-c_p _*≤ *π*_*p *_< 0, the initial concentration of the *p*^th ^molecular species is reduced, a situation that can be achieved by a variety of experimental techniques, such as RNA interference (RNAi). On the other hand, when *π_p _*> 0, the initial concentration of the *p*^th ^molecular species is increased, a situation that can be achieved by transfection or molecular injection.

Due to the enormous complexity of biological reaction networks, (1) is used to model a limited number of molecular interactions embedded within a larger and more complex system. Mass flow between the biochemical reaction system given by (1) and its surroundings complicates modeling. As a matter of fact, some molecular concentrations in the system may be influenced by unknown reactions, not modeled by (1), or by partially known reactions with reactants regulated by unknown biochemical mechanisms. To address this problem, we will assume that there is no appreciable mass transfer between the biochemical reaction system and its surroundings during the observation time interval T = [0, *t*_max_]. As a consequence, we can assume that (1) characterizes a *closed *biochemical reaction system within T. Moreover, we will assume that the system reaches *quasi-equilibrium *at some time t_* _≤ *t*_max_, after which its thermodynamic properties do not appreciably change for t_* _<*t *≤ *t*_max_. Note however that the quasi-equilibrium assumption does not necessarily imply that the biochemical reaction system will be at thermodynamic equilibrium after time *t*_max_, since mass transfer may take place at some time *t > t*_max_. Although we may be able to satisfy these assumptions by appropriately designed *synthetic *or *in vitro *biological experiments, the assumptions are certainly not satisfied *in vivo*. For this reason, we believe that future research must be focused on extending the approaches and techniques discussed in this paper to the case of *open *biochemical reaction systems.

### Measurements

We will now specify an appropriate model for the available measurements. We will assume that, by an appropriately designed experiment, we can obtain noisy measurements $y:=\{y_{n}^{\left(p\right)}(t_{q}),n\in N,p\in P,q\in Q\}$ and $\bar{y}:=\{y_{n}^{\left(p\right)}(t_{Q+1}),n\in N,p\in P\}$ of the concentrations of all molecular species in the unperturbed and perturbed systems at a limited number of distinct time points *t*_1 _<*t*_2 _< ⋯ <*t*_*Q *_<*t*_*Q*+1 _in T, where Q := {1, 2, ..., *Q*}. We will also assume that these measurements are related to the true concentrations $x_{n}^{\left(p\right)}(t_{q})$ by

(5) $$y_{n}^{\left(p\right)}(t_{q})=\ln\left[\epsilon_{n}^{\left(p\right)}(t_{q})x_{n}^{\left(p\right)}(t_{q})\right]=\ln x_{n}^{\left(p\right)}(t_{q})+\eta_{n}^{\left(p\right)}(t_{q}),\;n\in N,p\in P,$$

for *q *= 1, 2, ..., *Q *+ 1, where $\epsilon_{n}^{\left(p\right)}(t_{q})$ is a *multiplicative *random error factor and $\eta_{n}^{\left(p\right)}(t_{q}):=\ln\epsilon_{n}^{\left(p\right)}(t_{q})$. The assumption of multiplicative errors is common in most data acquisition procedures, such as DNA microarray-based genomics and mass spectrometry-based proteomics [28-30], whereas, the logarithm is used to obtain a convenient additive error model for the measurements.

In the following, we will assume that the biochemical reaction system, and all its perturbed versions, is sufficiently close to steady-state at time point *t*_*Q*+1_. We can justify this assumption by taking t_* _≤ *t*_*Q*+1 _≤ *t*_max _and by recalling our previous assumption that the biochemical reaction system is at thermodynamic quasi-equilibrium at times t_* _≤ *t *≤ *t*_max_. Our Bayesian analysis approach is based on data ***y***, whereas, we use the steady-state measurements $\bar{y}$ to derive a joint probability density function for the logarithms {ln(*k*_2*m*-1_/*k*_2*m*_), *m *∈ ℳ} of the equilibrium constants of the reactions needed for specifying the posterior density.

Finally, we will assume that the error components $\eta_{n}^{\left(p\right)}(t_{q})$ are statistically independent zero-mean Gaussian random variables. The Gaussian assumption is quite common in genomic problems and has been experimentally verified in some cases; e.g., see [31]. This assumption is usually justified by the central limit theorem and the premise that the errors are due to a large number of independent multiplicative error sources. We may attempt to justify the independence assumption between measurement errors by arguing that an error occurred in a particular measurement may only be due to the acquisition process used to obtain that measurement and, hence, it may not affect the error values of other measurements. In general, however, this is only a mathematically convenient assumption that may not be realistic. We experimentally demonstrate later that, at least for the example considered in this paper, the proposed estimation methodology is quite effective even in the case of non-Gaussian and correlated measurement errors. For simplicity, we finally assume equal error variances; i.e., we will assume that $\text{var}[\eta_{n}^{\left(p\right)}(t_{q})]=\sigma^{2}$, for every *n*, *p*, and *q*. This assumption is not crucial to our approach and can be relaxed if necessary.

### Bayesian model calibration

In this paper, we deal with the following problem: Given noisy concentration measurements ***y ***and $\bar{y}$, we want to calculate thermodynamically consistent estimates of the log-rate constants ***κ ***:= {*κ*_2*m*-1 _:= ln *k*_2*m*-1_, *κ*_2*m *_:= ln *k*_2*m*_, *m *∈ ℳ} of a closed biochemical reaction system, such that (2), initialized by (3), produce molecular concentrations $x_{n}^{\left(p\right)}(t)$ that "best" match (in some well-defined sense) the available measurements.

We should note here that it is convenient to estimate the logarithms of the rate constants instead of the constants themselves. By focusing on the logarithms, we can reduce the dynamic range of rate constant values and make their estimation numerically easier. To simplify our developments, we will assume that the initial concentrations {*c_n_, n *∈ N} and perturbations {*π_p_, p *∈ P} are known or have been estimated by an appropriate experimental procedure. When this is not true, these quantities must be treated as unknown parameters and estimated from data, together with the rate constants, provided that a sufficient amount of data is available to allow reliable estimation.

Given data ***y***, the objective of Bayesian analysis is to evaluate the *posterior *probability density function *p*(***κ ***| ***y***), which summarizes our belief about the log-rate constants ***κ ****after *the data ***y ***have been collected. It can be shown [see Equations (S-1.4) and (S-1.5) in Additional file 1] that

(6) p(κ|y)∝p(y|κ)∫p(κ|z)p(z)dz,

where *p *∝ *q *denotes that *p *is proportional to *q*, and

(7) $$p(y|\kappa )=\int p(y|\kappa ,\sigma^{2})p(\sigma^{2}|\kappa )d\sigma^{2},$$

with ***z ***= {*z_m_, m *∈ ℳ} being the set of log-equilibrium constants of the reactions, defined by

(8) $$z_{m}:=\ln\frac{k_{2m-1}}{k_{2m}}=\kappa_{2m-1}-\kappa_{2m},\;\text{for}\;m\in ℳ.$$

Note that the prior density of the log-rate constants ***κ ***depends on ***z***. For this reason, we view ***z ***as a set of random hyperparameters (in Bayesian analysis, parameters used to specify prior densities are known as hyperparameters), specified by means of the prior density *p*(***z***).

The posterior density *p*(***κ ***| ***y***) takes into account our prior belief about the rate constant values and the data formation process, summarized by the prior density *p*(***z***) of the log-equilibrium constants, the conditional prior density *p*(***κ ***| ***z***) of the log-rate constants given the log-equilibrium constants, the conditional probability density *p*(*σ*^2 ^| ***κ***) of the error variance given the log-rate constants, and the likelihood *p*(***y ***| ***κ***, *σ*^2^). However, the posterior density is hard to interpret, especially in high-dimensional problems that involve many parameters, such as the problem we are dealing with here. As a consequence, the main objective of Bayesian analysis is to produce numerical information that can be effectively used to summarize the posterior density and simplify the task of statistical inference to the extent possible. Typical summaries include measures of location and scale of the posterior, which are used to produce estimates for the parameter values and to evaluate the accuracy of such estimates, respectively.

It is clear from (6) that, to evaluate the posterior *p*(***κ ***| ***y***), we need to compute the "effective" prior density ∫ *p*(***κ ***| ***z***)*p*(***z***) *d****z ***as well as the "effective" likelihood ∫ *p*(***y ***| ***κ***, *σ*^2^)*p*(*σ*^2 ^| ***κ***)*dσ*^2^. To do so, we must specify the aforementioned densities *p*(*σ*^2 ^| ***κ***), *p*(***z***), *p*(***κ ***| ***z***), and *p*(***y ***| ***κ*, ***σ*^2^). We discuss this problem next.

### Prior density of error variance

In general, it is difficult to derive an informative prior probability density function *p*(*σ*^2 ^| ***κ***) for the error variance. To deal with this problem, we assume here that the error variance is independent of the rate constants; i.e., we assume that *p*(*σ*^2 ^| ***κ***) = *p*(*σ*^2^). Moreover, we assume that *σ*^2 ^follows an inverse gamma distribution, in which case

(9) $$p(\sigma^{2})=\frac{b^{\alpha }}{\Gamma (\alpha )}{\left(\sigma^{2}\right)}^{-(\alpha +1)}e^{-b/\sigma^{2}},$$

for two parameters *α, b >*0.

The independence assumption between *σ*^2 ^and ***κ ***is reasonable, in view of the fact that the errors are mainly due to the experimental methodology used to obtain the measurements, whereas, the rate constants are due to biophysical principles underlying the biochemical reaction system. On the other hand, the choice given by (9) has been well-justified in Bayesian analysis. In fact, the inverse gamma distribution is the conjugate prior for the variance of additive Gaussian errors [13]. Conjugate priors are common in Bayesian analysis, since they often lead to attractive analytical and computational simplifications. Note that **E**[*σ*^2^] = *b/*(*α *- 1) and var[*σ*^2^] = {**E**[*σ*^2^]}^2^/(*α *- 2) = *b*^2^/[(*α *- 1)^2^(*α *- 2)], for *α >*2. Therefore, the parameters *α, b *control the location and scale of the inverse gamma distribution given by (9). We illustrate this prior in Figure S-1.3 of Additional file 1. In the following, we treat *α *and *b *as hyperparameters with known values. For a practical method to determine these values, the reader is referred to Additional file 1.

### Prior density of log-equilibrium constants

Before we consider the problem of specifying a prior density for the log-equilibrium constants ***z***, we first investigate how much information about ***z ***can be extracted from measurements.

It is a direct consequence of thermodynamic analysis that, at steady-state, the net flux of each reaction in a closed biochemical reaction system must be zero. This implies that

(10) $$k_{2m-1}\prod_{{}_{n\in N}}{\left[{\bar{x}}_{n}^{\left(p\right)}\right]}^{\nu_{nm}}=k_{2m}\prod_{{}_{n\in N}}{\left[{\bar{x}}_{n}^{\left(p\right)}\right]}^{{\nu^{\prime }}_{nm}},\;\text{for all }m\in ℳ,p\in P,$$

by virtue of (4), where $\left\{{\bar{x}}_{n}^{\left(p\right)}>0,n\in N\right\}$ are the stationary concentrations when the initial concentration of the *p*^th ^molecular species is perturbed (thermodynamic analysis dictates that these concentrations must be nonzero, provided that the initial concentrations are nonzero). As a matter of fact, (10) is equivalent to the following constraints on the reaction rate constants (see Additional file 1):

(11) $$\prod_{m\in ℳ}{\left(\frac{k_{2m-1}}{k_{2m}}\right)}^{r_{m}}=1,\;\text{for all }r\in \text{null}(S),$$

known as Wegscheider conditions [14,15], where *r_m _*is the *m*^th ^element of the *M *× 1 vector ***r***, S is the *N × M *stoichiometry matrix of the biochemical reaction system with elements *s_nm_*, and null(S) is the null space of S. As a consequence, for a biochemical reaction system to be physically realizable, it is required that the reaction rates satisfy the thermodynamically imposed Wegscheider conditions.

From (8) and (10), note that

(12) $$z_{m}=\frac{1}{P+1}\sum_{p\in P}\sum_{n\in N}s_{nm}\ln{\bar{x}}_{n}^{\left(p\right)},\;\text{for all }m\in ℳ.$$

By employing (5) and (12), we can show that $z_{m}={\tilde{y}}_{m}-{\tilde{\eta }}_{m}$, where

(13) $${\tilde{y}}_{m}:=\frac{1}{P+1}\sum_{p\in P}\sum_{n\in N}s_{nm}y_{n}^{\left(p\right)}(t_{Q+1})\;\text{and}\;{\tilde{\eta }}_{m}:=\frac{1}{P+1}\sum_{p\in P}\sum_{n\in N}s_{nm}\eta_{n}^{\left(p\right)}(t_{Q+1}).$$

Using this result and some straightforward algebra (see Additional file 1), we can show that, given $\tilde{y}:=\{{\tilde{y}}_{m},m\in ℳ\}$, which can be calculated from the measurements $\bar{y}=\{y_{n}^{\left(p\right)}(t_{Q+1}),n\in N,p\in P\}$ of the steady-state molecular concentrations and (13), we can construct the posterior density $p(z|\tilde{y})$ of ***z ***by

(14) $$p(z|\tilde{y})\propto {\left[\frac{2b}{P+1}+{\left(z-\tilde{y}\right)}^{T}{ℍ}^{-1}(z-\tilde{y})\right]}^{-(M/2+\alpha )},$$

where ℍ is an *M *× *M *matrix with elements $h_{mm^{\prime }}=\sum_{n\in N}s_{nm}s_{nm^{\prime }}$, and *α*, *b *are the two hyperparameters associated with the prior density of the measurement variance, given by (9).

The previous result suggests that we may be able to use $p(z|\tilde{y})$ as an informative prior for the log-equilibrium constants ***z***; i.e., we may be able to replace *p*(***z***) by $p(z|\tilde{y})$ in (6). At a first glance, this idea may not seem appropriate. However, it perfectly agrees with the fact that, in Bayesian analysis, hyperparameters are often estimated directly from data [13]. Since we have shown here that steady-state measurements can be effectively used to construct the entire posterior probability density function of ***z***, it seems reasonable to use this posterior as a prior density for ***z***. Note however that, by replacing *p*(***z***) with $p(z|\tilde{y})$ in (6), we must make sure that $\tilde{y}$ is independent of ***y ***(see Additional file 1). Otherwise, our choice for *p*(***z***) may not lead to a proper posterior density *p*(***κ ***| ***y***) (i.e., it may not lead to a density that is finite for all ***y***). Note that the independence of $\tilde{y}$ and ***y ***is assured by the independence between the measurement errors $\left\{\eta_{n}^{\left(p\right)}(t_{Q+1}),n\in N,p\in P\right\}$ and $\left\{\eta_{n}^{\left(p\right)}(t_{q}),n\in N,p\in P,q\in Q\right\}$.

An important observation here is that evaluation of $p(z|\tilde{y})$, given by (14), may not be possible, since the matrix ℍ may not be invertible. We can address this problem by decorrelating ***z ***using the singular value decomposition (SVD) of matrix ℍ. As a consequence, we obtain $ℍ=U_{0}D_{0}U_{0}^{T}$, where $D_{0}$ is an invertible diagonal matrix containing the nonzero singular values of ℍ, and $U_{0}$ is an appropriately constructed matrix (see Additional file 1 for details). In this case, instead of using (14) for $p(z|\tilde{y})$, we must use

(15) $$p(z|\tilde{y})\propto {\left[\frac{2b}{P+1}+{\left(z-\tilde{y}\right)}^{T}U_{0}D_{0}^{-1}U_{0}^{T}(z-\tilde{y})\right]}^{-\alpha },$$

which we can always evaluate, since matrix $D_{0}$ is invertible.

### Prior density of log-rate constants

To specify the (conditional) prior density *p*(***κ ***| ***z***) of the log-rate constants of a biochemical reaction system, we will first derive a prior probability density function *p*(*κ*_2*m*-1_) for the log-rate constant of the *m*^th ^forward reaction. To do so, we use the well-known Arrhenius formula of chemical kinetics [21] and set *k*_2*m*-1 _= *α*_*m *_exp{-*E_m_/k_B_T*}, where *α*_*m *_is the prefactor, *E*_*m *_is the activation energy of the reaction, *k*_*B *_is the Boltzmann constant (*k*_*B *_= 1.3806504 × 10^-23^J/K), and *T *is the temperature. Unfortunately, we cannot predict the values of the prefactor and activation energy precisely. To deal with this problem, we set $\alpha_{m}=\alpha_{m}^{0}\exp\{g_{m}\}$ and $E_{m}=E_{m}^{0}+U_{m}$, where $\alpha_{m}^{0},E_{m}^{0}$ are the predictable portions of the prefactor and activation energy, respectively, and *g_m_*, *U_m _*are two random variables that model the unpredictable portions of these quantities. In the Additional file 1, we argue that it is reasonable to model *g_m _*as a zero-mean Gaussian random variable with standard deviation *λ_m_*, and *U_m _*is an exponential random variable with mean and standard deviation $k_{B}T_{m}^{*}$, where $T_{m}^{*}$ is a temperature larger than *T *. As a consequence, we obtain the following prior density for the log-rate constant *κ*_2*m*-1 _of the *m*^th ^forward reaction [see Equation (S-1.31) in Additional file 1]:

(16) $$p(\kappa_{2m-1})=\frac{e^{\lambda_{m}^{2}/2\tau_{m}^{2}}}{2\tau_{m}}\text{erfc}\left[\frac{1}{\sqrt{2}}\left(\frac{\lambda_{m}}{\tau_{m}}+\frac{\kappa_{2m-1}-\kappa_{m}^{0}}{\lambda_{m}}\right)\right]e^{(\kappa_{2m-1}-\kappa_{m}^{0})/\tau_{m}},$$

where $\tau_{m}:=T_{m}^{*}/T>1$, $\kappa_{m}^{0}:=\ln\alpha_{m}^{0}-E_{m}^{0}/k_{B}T$, and erfc[·] is the complementary error function. We illustrate this prior in Figure S-1.1 of Additional file 1.

Basic thermodynamic arguments (see Additional file 1) imply that *z_m_*, defined by (8), is a constant characteristic to the *m*^th ^reaction. Since *κ*_2*m *_= *κ*_2*m*-1 _- *z*_*m*_, this implies that the rate constants *κ*_2*m *_and *κ*_2*m*-1 _are two dependent random variables, given *z_m_*, with joint probability density *p*(*κ*_2*m*_, *κ*_2*m*-1 _| *z*_*m*_) = *δ*(*κ*_2*m *_- *κ*_2*m*-1 _+ *z*_*m*_)*p*(*κ*_2*m*-1_), where *δ*(·) is the Dirac delta function [see Equation (S-1.37) in Additional file 1]. By assuming that the reaction rate constants of different reactions are mutually independent given the *z*'s (which is reasonable if we assume that all common factors affecting these rates, such as temperature and pressure, are kept fixed), we obtain

(17) $$p(\kappa |z)=\prod_{m\in ℳ}\delta (\kappa_{2m}-\kappa_{2m-1}+z_{m})p(\kappa_{2m-1}).$$

Equations (16) and (17) provide an analytical form for the prior density of the log-rate constants. To use this expression, we must determine appropriate values for $\phi :=\{\kappa_{m}^{0},\tau_{m},\lambda_{m},m\in ℳ\}$, which can be treated as hyperparameters. Although we could treat ***ϕ ***as random, we will choose here known values for these parameters. This is motivated by the fact that ***ϕ ***determines the location and scale of the prior densities of the forward rate constants; see Figure S-1.1 in Additional file 1. In certain problems of interest, there might be enough information to determine possible ranges for the forward rate constant values. As a consequence, we can use this information, together with an appropriate procedure, to effectively determine values for ***ϕ***. The reader is referred to Additional file 1 for details on how to do so.

### Effective likelihood

Calculating the effective likelihood *p*(***y ***| ***κ***), given by (7), is straightforward. From (5), (7), and (9), we can show that

(18) $$p(y|\kappa )\propto \int \frac{1}{\sigma^{N(P+1)Q+2(\alpha +1)}}\exp\left\{-\frac{\varphi (\kappa ,y)}{2\sigma^{2}}\right\}d\sigma^{2},$$

where

(19) $$\varphi (\kappa ,y):=2b+\sum_{n\in N}\sum_{p\in P}\sum_{q\in Q}{\left[y_{n}^{\left(p\right)}(t_{q})-\ln x_{n}^{\left(p\right)}(t_{q})\right]}^{2}.$$

By setting *ξ *= *φ*(***κ,y***)/2*σ*^2 ^in (18), we obtain

(20) p(y|κ)∝[φ(κ,y)]−α−N(P+1)Q/2∫ξα+N(P+1)Q/2−1e−ξdξ ∝[φ(κ,y)]−α−N(P+1)Q/2.

Note that evaluating *φ*(***κ,y***) at given values of ***κ ***and ***y ***requires integration of the system of ordinary differential equations (2).

### Posterior density

Our previous developments lead finally to an analytical formula for the posterior density *p*(***κ ***| ***y***) of the log-rate constants. Indeed, (6), (15), (17), (19), and (20), lead to

(21) $$p(\kappa |y)\propto \frac{\omega (\kappa )}{{\left[\psi (\kappa ,y)\right]}^{\alpha }{\left[\varphi (\kappa ,y)\right]}^{\beta }},$$

with

(22) $$\begin{array}{l} \omega (\kappa )=\prod_{m\in ℳ}\text{erfc}\left[\frac{1}{\sqrt{2}}\left(\frac{\lambda_{m}}{\tau_{m}}+\frac{\kappa_{2m-1}-\kappa_{m}^{0}}{\lambda_{m}}\right)\right]e^{\kappa_{2m-1}/\tau_{m}}\\ \psi (\kappa ,y)=\frac{2b}{P+1}+\sum_{m\in ℳ}\sum_{m'\in ℳ}\theta_{mm^{\prime }}(\kappa_{2m-1}-\kappa_{2m}-{\tilde{y}}_{m})(\kappa_{2m^{\prime }-1}-\kappa_{2m^{\prime }}-{\tilde{y}}_{m^{\prime }})\\ \varphi (\kappa ,y)=2b+\sum_{n\in N}\sum_{p\in P}\sum_{{}_{q\in Q}}{\left[y_{n}^{\left(p\right)}(t_{q})-\ln x_{n}^{\left(p\right)}(t_{q})\right]}^{2}\\ \beta =\alpha +N(P+1)Q/2, \end{array}$$

where θ*_mm' _*are the elements of matrix $U_{0}D_{0}^{-1}U_{0}^{T}$ obtained from the SVD decomposition of $S^{T}S$, and ${\tilde{y}}_{m}$ is given by (13).

Note that the posterior density of the log-rate constants is a compromise between the prior and the likelihood. The prior terms *ω*(***κ***) and *ψ*(***κ***, ***y***) penalize log-rate values that do not fit well with available a-priori information, whereas, the likelihood term *φ*(***κ***, ***y***) penalizes log-rate values that produce concentration dynamics which deviate appreciably from measurements. As the number *N*(*P *+ 1)*Q *of available measurements increases, this compromise is controlled to a greater extent by the data through the factor *φ*(***κ***, ***y***).

A problem arises with the posterior density *p*(***κ ***| ***y***), given by (21) and (22), since nonzero probabilities may be assigned to thermodynamically infeasible log-rate constants. A Bayesian analyst might argue that we have correctly done our job by formulating the problem as we did and that it is the data which will rule out the possibility that our biochemical reaction system can be characterized by thermodynamically infeasible parameters. However, we choose to trust thermodynamics far more than we would trust noisy data and appropriately modify the posterior density based on our knowledge that the kinetic parameters must satisfy the Wegscheider conditions given by (11).

By using the Wegscheider conditions, we can decompose the 2*M *log-rate constants ***κ ***into two mutually exclusive sets: *M *+ *M*_1 _"free" log-rate constants ***κ***_*f *_and *M *− *M*_1 _"dependent" log-rate constants ***κ***_*d*_, where $M_{1}=\text{rank}(S)$ (see Additional file 1). Although parameters ***κ***_*f *_can take any value, parameters ***κ***_*d *_must be equal to $W\kappa_{f}$ for the Wegscheider conditions to be satisfied, where W is an appropriately defined matrix. One way to incorporate the constraint $\kappa_{d}=W\kappa_{f}$ into our Bayesian analysis problem is to treat it as prior information and apply it on the prior density of the unconstrained problem. This principle forms the basis of an attractive strategy for incorporating constraints into Bayesian analysis, known as encompassing prior approach (EPA) [32]. By following EPA, we can replace the previously discussed encompassing "effective" prior density ∫ *p*(***κ ***| ***z***)*p*(***z***)*dz *by the following probability density function:

(23) $$p_{w}(\kappa_{f},\kappa_{d})\text{ :}=\frac{\delta (\kappa_{d}-W\kappa_{f})\int p(\kappa_{f},\kappa_{d}|z)p(z)dz}{∭\delta (\kappa_{d}-W\kappa_{f})p(\kappa_{f},\kappa_{d}|z)p(z)dzd\kappa_{f}d\kappa_{d}},$$

where *δ *is the Dirac delta function. Clearly, this density assigns zero probability to kinetic parameters that do not satisfy the Wegscheider conditions, since $\delta (\kappa_{d}-W\kappa_{f})=0$, if $\kappa_{d}\neq W\kappa_{f}$. Note now that the log-rate constants ***κ***_*d *_are of no immediate interest, since their values can be determined as soon as the values of the log-rate constants ***κ***_*f *_have been estimated. As a consequence, we can treat ***κ***_*d *_as "nuisance" parameters and integrate them out of the problem [13]. This integration, together with the updated prior density we presented above, leads to the following *marginal *posterior density of the log-rate constants ***κ***_*f *_:

(24) $$p_{w}(\kappa_{f}|y)\propto \int \delta (\kappa_{d}-W\kappa_{f})p(\kappa_{f},\kappa_{d}|y)d\kappa_{d}=p(\kappa_{f},W\kappa_{f}|y).$$

Clearly, the values of the marginal posterior *p_W _*(***κ***_*f *_| ***y***) are proportional to the corresponding values of the original posterior density *p*(***κ***_*f *_, *κ*_*d *_| ***y***) over the thermodynamically feasible region of the parameter space, given by the hyperplane $\kappa_{d}=W\kappa_{f}$. In the following, we will base our Bayesian analysis approach on *p_W _*(***κ***_*f *_| ***y***).

### Computing the posterior mode

In a Bayesian setting, we use the location of the posterior density over the parameter space to provide an estimate of the unknown parameter values. Typically, two measures of location are employed, namely the mode and the mean of the posterior. The posterior mean minimizes the mean-square error between the estimated and true parameters, whereas, the posterior mode is more likely to produce dynamics that closely resemble the true dynamics (see Additional file 1 for why this is true). We note here that the main objective of parameter estimation in biochemical reaction systems is not necessarily to determine parameter values that are "close" to the true values (e.g., in the mean square sense) but to obtain appropriate values for the rate constants so that the resulting molecular concentration dynamics closely reproduce the dynamics observed in the true system [33]. As a consequence, we choose the posterior mode as our parameter estimator.

The posterior log-density ln*p_W _*(***κ***_*f *_| ***y***) is usually not concave, especially when a limited amount of highly noisy data ***y ***is available. As a consequence, there is no optimization algorithm that can find the posterior mode in a finite number of steps. A method to address this problem would be to randomly sample the parameter space at a predefined (and usually large) number of points and use these points to initialize an optimization algorithm, such as the simultaneous perturbation stochastic approximation (SPSA) algorithm discussed in the Additional file 2. We can then calculate the parameters and the associated values of ln*p_W _*(***κ***_*f *_| ***y***) obtained by each initialization after a set number of optimization steps, and declare the parameters associated with the highest log-posterior value as being the desired mode estimates.

Unfortunately, SPSA (and as a matter of fact any other appropriate optimization algorithm) is computationally costly, especially in the case of large biochemical reaction systems. Therefore, using SPSA in the previous multi-seed strategy may result in a computationally prohibitive approach for finding the posterior mode. In order to reduce computations, we may choose only a small number of initial points that we believe are sufficiently proximal to the posterior mode. Two such points might be the prior and posterior means. As a matter of fact, as the data sample size tends to infinity, we expect that the posterior mean will coincide with the posterior mode, since, under suitable regularity conditions, the posterior density converges asymptotically to a Gaussian distribution [12,34]. This simple idea leads to the sequential maximization-expectation-maximization (MEM) algorithm we discuss in the Additional file 2. According to this algorithm, we perform a relatively small number of SPSA iterations, initialized by the prior mode, to obtain a posterior mode estimate ${\hat{\kappa }}_{f,1}^{\text{mode}}$. We then use an MCMC algorithm, initialized by ${\hat{\kappa }}_{f,1}^{\text{mode}}$, to obtain an estimate of the posterior mean ${\hat{\kappa }}_{f}^{\text{mean}}$. Subsequently, we perform another set of SPSA iterations, initialized by ${\hat{\kappa }}_{f}^{\text{mean}}$, to obtain the posterior mode estimate ${\hat{\kappa }}_{f,2}^{\text{mode}}$. We finally set ${\hat{\kappa }}_{f}^{\text{mode}}$ to be the log-rate constants that produce the maximum posterior value during all SPSA and MCMC iterations, and set the optimal estimate $\hat{\kappa }$ of the log-rate constants ***κ ***equal to $\left\{{\hat{\kappa }}_{f}^{\text{mode}},W{\hat{\kappa }}_{f}^{\text{mode}}\right\}$.

### Estimation accuracy

One way to quantify the accuracy of the posterior mode estimate ${\hat{\kappa }}_{f}^{\text{mode}}$ of a "free" log-rate constant *κ_f _*is to calculate and report the root mean square error (RMSE), given by

(25) $$\epsilon_{\text{RMSE}}({\hat{\kappa }}_{f}^{\text{mode}})=\sqrt{\text{E}[{\left(\kappa_{f}-{\hat{\kappa }}_{f}^{\text{mode}}\right)}^{2}|y]}={\left\{\int {\left(\kappa_{f}-{\hat{\kappa }}_{f}^{\text{mode}}\right)}^{2}p_{W}(\kappa_{f}|y)d\kappa_{f}\right\}}^{1/2}.$$

A small value of ϵ_RMSE _provides us with confidence that the estimated value of that constant is accurate. On the other hand, the estimate may be perceived as inaccurate if ϵ_RMSE _is exceedingly large.

Another useful metric for evaluating estimation accuracy is $D:=\ln\det[V]/(M+M_{1})$, where det[V] is the determinant of the posterior covariance matrix $V=\text{E}\left[(\kappa_{f}-{\hat{\kappa }}_{f}^{\text{mode}}){\left(\kappa_{f}-{\hat{\kappa }}_{f}^{\text{mode}}\right)}^{T}|y\right]$. Note that *D *is the average of the log-eigenvalues of V and is related to the well-known *D*-optimal criterion used in experimental design [27]. We can use *D *to quantify the overall accuracy of a model calibration result, with smaller values of *D *indicating better overall accuracy.

Note that the RMSE's ϵ_RMSE _can be computed from the diagonal elements of V. It turns out the we can approximate ϵ_RMSE _and *D *from an estimate $\hat{V}$ of the posterior covariance matrix V obtained during the second (MCMC) phase of the proposed MEM algorithm (see Additional file 2 for details).

When the true values ***κ***^true ^of the log-rate constants are known (which is the case when we use simulated data to evaluate the performance of the proposed Bayesian analysis approach, as we do in this paper), we can provide a more direct evaluation of estimation performance. As we have mentioned previously, calculating a measure of "closeness" (such as the square error) between the estimated and true parameter values may not be quite appropriate here. Since, in reality, our objective is to estimate the rate constant values so that the biochemical reaction system produces dynamics that closely match the true molecular dynamics, it may be more appropriate to use, as measures of estimation performance, the following *median *and *maximum *absolute error criteria:

(26) $$\begin{array}{l} \epsilon_{\text{MED-AE}}=\underset{n\in N,p\in P}{\text{med}}\left\{\frac{\int_{T}\left|{\hat{x}}_{n}^{\left(p\right)}(t)-x_{n}^{\left(p\right)}(t)\right|dt}{\int_{T}x_{n}^{\left(p\right)}(t)dt}\right\}\\ \epsilon_{\text{MAX-AE}}=\underset{n\in N,p\in P}{\text{max}}\left\{\frac{\int_{T}\left|{\hat{x}}_{n}^{\left(p\right)}(t)-x_{n}^{\left(p\right)}(t)\right|dt}{\int_{T}x_{n}^{\left(p\right)}(t)dt}\right\}, \end{array}$$

where $\left\{x_{n}^{\left(p\right)}(t),t\in T\right\}$ and $\left\{{\hat{x}}_{n}^{\left(p\right)}(t),t\in T\right\}$ are the true and estimated dynamics of the *n*^th ^molecular species under the *p*^th ^perturbation, produced by the biochemical reaction system with log-rate constants ***κ***^true ^and $\hat{\kappa }=\{{\hat{\kappa }}_{f}^{\text{mode}},W{\hat{\kappa }}_{f}^{\text{mode}}\}$, respectively. Clearly, ϵ_MED-AE _and ϵ_MAX-AE _provide measures of closeness between the estimated molecular responses $\left\{{\hat{x}}_{n}^{\left(p\right)}(t),t\in T,n\in N,p\in P\right\}$ and the true molecular responses $\left\{x_{n}^{\left(p\right)}(t),t\in T,n\in N,p\in P\right\}$, normalized by the corresponding true integrated responses $\left\{\int_{T}x_{n}^{\left(p\right)}(t)dt,n\in N,p\in P\right\}$. Normalization is required in order to make sure that no one species dominates the error values more than any other. Finally, note that half of the normalized absolute errors will be between 0 and ϵ_MED-AE_, whereas, the remaining half will be between ϵ_MED-AE _and ϵ_MAX-AE_.

## Results/Discussion

**Figure 1:**
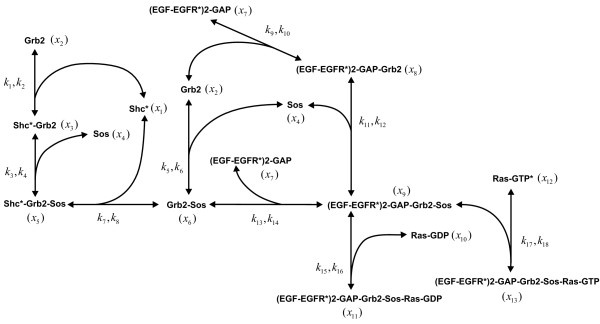
**A subset of the EGF/ERK signal transduction pathway model proposed in **[35]. The biochemical reaction system is comprised of *N *= 13 molecular species that interact through *M *= 9 reactions. Bayesian analysis is focused on estimating the values of the 18 rate constants associated with the reactions.

To illustrate key aspects of the previous Bayesian analysis methodology, we now consider a numerical example based on a subset of a well-established model of the EGF/ERK signal transduction pathway proposed by Schoeberl *et al. *[35]. This model corresponds to an open biochemical reaction system, since it contains irreversible reactions as well as reactions governed by Michaelis-Menten kinetics that involve molecular species not included in the model. We extract a closed subset of the Schoeberl model by choosing the largest connected section that contains only reversible reactions governed by mass action kinetics. The resulting biochemical reaction system is depicted in Figure 1 and is comprised of *N *= 13 molecular species that interact through *M *= 9 reversible reactions. Of course, we could attempt to generate a closed biochemical reaction system for the entire EGF/ERK signaling pathway, by including all relevant molecular species not considered by the Schoeberl model (e.g., ADP, ATP, intermediate forms in catalyzed reactions, etc.). However, since we are only interested in demonstrating the potential and key properties of our Bayesian analysis methodology, we found this to be unnecessary. We feel that the biochemical reaction system depicted in Figure 1 leads to a sufficiently rich numerical example that serves the main purpose of this section well.

In specifying the model depicted in Figure 1, we must provide three sets of physically reasonable values: true rate constant values, initial concentrations, and experimentally feasible perturbations to the initial concentrations. Published values for the reaction rate constants associated with our example are given in Equation (S-3.1) of Additional file 3. However, these values do not correspond to a thermodynamically feasible biochemical reaction system, since they do not satisfy the Wegscheider conditions, given by (11). We should point out here that this is a common problem in systems biology. Reaction rate values are usually amalgamated from various independent sources in the literature, so it is highly unlikely that these values will correspond to a thermodynamically feasible biochemical reaction system. As a consequence, it is desirable to develop a method that uses published values for the reaction rate constants and calculates an appropriate set of thermodynamically feasible values that can be considered as the "true" parameter values. In Additional file 3, we calculate "true" values for the log-rate constants by using a linear least squares approach to project the published values onto the thermodynamically feasible hyperplane. The resulting "true" values are given in Equation (S-3.3) of Additional file 3.

Regarding the initial concentrations, we use the values specified in [35,36], with two minor modifications. First, molecular species with zero initial concentrations are modified to have a small number of molecules present. We do this to accommodate the fact that, in a real cellular system, these molecular species are constitutively expressed. The second modification comes from the fact that we are no longer modeling the entire EGF/ERK signaling cascade and, therefore, we must account for the upstream EGF stimulus. To take this into account, we increase the initial concentration of the most upstream molecular species in our model, namely (EGF-EGFR*)2-GAP. The initial concentrations used are given by Equation (S-3.4) in Additional file 3.

To specify appropriate perturbations to the initial molecular concentrations, note that molecular complexes, such as dimers, trimers, etc., are far more difficult to perturb than simple monomeric molecular species. For this reason, we focus our perturbation efforts on Shc*, Grb2, and Sos. Since Shc* is commercially available in a purified and quantified form, we will assume that we can increase its initial concentration by a factor of 100 using molecular injection. We will also assume that we can perturb Grb2 and Sos by RNAi, resulting in a decrease in their initial concentrations by a factor of 100. Thus, we set *π*_1 _= 99*c*_1_, *π*_2 _= -.99*c*_2_, and *π*_4 _= -.99*c*_4_.

To avoid specifying different hyperparameter values for the prior densities of the forward log-rate constants, we assume here that all densities share the same known values {*κ*^0^, *τ, λ*}, where *κ*^0 ^= -5.1010, *τ *= 1.8990, and *λ *= 0.7409, whereas, we set *α *= 3 and *b *= 1 for the hyperparameters of the prior density of the variance *σ*^2 ^of the measurement errors. These choices correspond to the prior densities depicted in Figure S-1.2(a) and Figure S-1.3(a) in Additional file 1. We implement our Bayesian analysis approach using the MEM algorithm described in Additional file 2, with *I *= 5,000 SPSA iterations in each maximization step and a total of *L *= 50,000 MCMC iterations in the expectation step. Finally, we observe the biochemical reaction system within a time period of 1 min.

**Figure 2:**
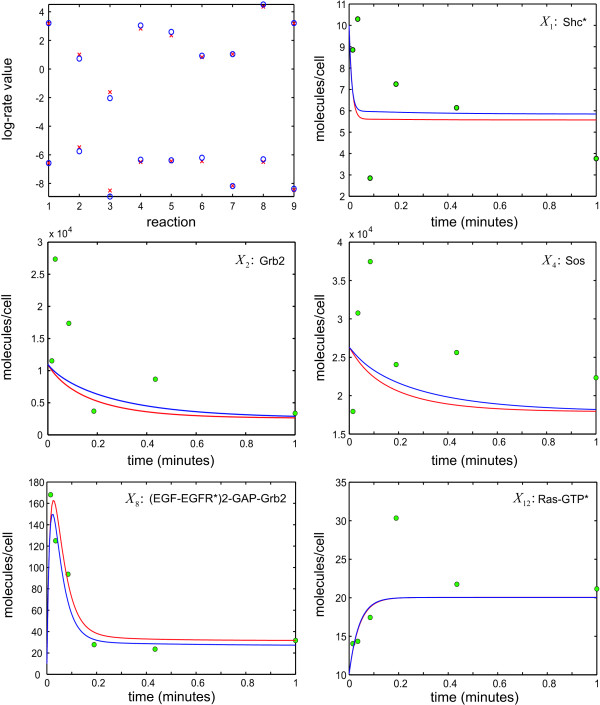
**True (red) vs. estimated (blue) log-rate values and selected molecular dynamics in the unperturbed biochemical reaction system depicted in Figure 1**. The results are based on measuring the dynamics in the unperturbed and perturbed systems at *Q *= 6 logarithmically-spaced time points (green circles). Perturbations are applied on the initial concentrations of Shc*, Grb2, and Sos, one at a time. The measurement errors are i.i.d. zero-mean Gaussian with standard deviation *σ *= 0.3.

In Figure 2, we depict a typical result obtained by the proposed Bayesian analysis algorithm. In this figure, we compare the estimated log-rate values (blue) with the thermodynamically consistent true log-rate values (red) as well as the corresponding concentration dynamics of selected molecular species in the unperturbed biochemical reaction system. We have obtained these results by measuring the concentration dynamics in the unperturbed and perturbed systems at *Q *= 6 logarithmically-spaced time points (green circles), with the measurements being corrupted by independent and identically distributed (i.i.d.) zero-mean Gaussian noise with standard deviation *σ *= 0.3. Moreover, we summarize the estimated posterior RMSE values, given by (25), in Table 1. Finally, the calculated median and maximum absolute error values, given by (26), are 3.03 × 10^-2 ^and 1.68 × 10^-1^, respectively.

**Table 1:** Estimated posterior RMSE values for the case of i.i.d. zero-mean Gaussian errors with standard deviation *σ *= 0.3. Logarithmic sampling is used with *Q *= 6.

*κ*_1_	*κ*_3_	*κ*_5_	*κ*_7_	*κ*_9_	*κ*_11_	*κ*_13_	*κ*_15_	*κ*_17_
0.2414	0.1578	0.1838	0.2950	0.1426	0.1683	0.0968	0.4474	0.1484

*κ*_2_	*κ*_4_	*κ*_6_	*κ*_8_	*κ*_10_	*κ*_12_	*κ*_14_	*κ*_16_	*κ*_18_

0.2594	0.2095	0.1704	-	0.2124	0.2136	-	0.5093	0.0494

The log-rate constants *κ*_8 _and *κ*_14 _are "dependent" variables. Therefore, no RMSE values are reported for these variables.

The concentration dynamics produced by the estimated rate constant values match well the dynamics produced by the true values. As a matter of fact, the calculated median and maximum absolute error values imply that half of the relative integrated absolute error values between the estimated and true concentration dynamics (across all molecular species and all applied perturbations) are smaller than 3.03%, whereas, the remaining values are between 3.03% and 16.8%. On the other hand, the estimated posterior RMSE values summarized in Table 1 indicate a high probability that, given the concentration measurements, the log-rate values will lie within a relatively small region around the corresponding posterior mode values.

We expect that, in general, by selecting appropriate perturbations and by increasing the number of concentration data collected during an experiment, we can improve estimation accuracy. However, how can one know if the right perturbations have been applied on the biochemical reaction system and if enough data has been collected in a practical situation? Inspection of RMSE values can provide an answer to these important questions. If the estimated RMSE values of the log-rate constants of many reactions are large, it may be worth collecting additional data by increasing *P *and *Q*. Additional data can improve estimation accuracy by shrinking the RMSE values to a size that indicates an acceptable degree of uncertainty. However, if the biochemical reaction system is insensitive to a given kinetic parameter, then the RMSE associated with that reaction may remain large even as the quality of data improves. Therefore, additional data should only be collected when the RMSE values are large and sensitivity analysis indicates that the values of the rate constants associated with these RMSE values appreciably affect the system dynamics.

The RMSE values do not provide a global measure of estimation accuracy, since some parameters may have small RMSE values and some may have large values. To address this problem, we may instead employ the *D*-optimal criterion as a measure of estimation accuracy. As a matter of fact, we can effectively use the *D*-optimal criterion as a guide for selecting appropriate perturbations and for determining the data sampling scheme we must use in order to increase estimation accuracy. In Table 2, for example, we summarize estimated values of *D*, for the case of uniform and logarithmic sampling, calculated for different values of *Q*. Clearly, the sampling scheme used may appreciably affect estimation performance. For each value of *Q*, uniform sampling results in higher values of *D *than logarithmic sampling. As a consequence, we must use logarithmic sampling over uniform sampling, since the former may produce better estimation accuracy than the latter. This is expected, since uniform sampling may result in measuring steady-state concentrations much more often than (short-lived) transient concentrations. On the other hand, logarithmic sampling may be used to gather valuable information about the transient behavior of a biochemical reaction system while placing less emphasis on its steady-state dynamics (which only provide information about the equilibrium constants of the underlying reactions). The results depicted in Table 2 also suggest an appropriate value for *Q*. If our goal is to find the smallest value Q* of *Q *(an objective dictated by the high cost of experimentally measuring molecular concentrations) which results in a value of *D *that is no less than, say 5%, of the value obtained when *Q *= Q* - 1, then we must set Q* = 6.

**Table 2:** Estimated values of the *D*-optimal criterion for uniform and logarithmic sampling schemes.

*Q*	uniform	logarithmic	% change
2	-1.7697	-2.3500	-
3	-2.0030	-3.4287	45.90%
4	-2.3752	-3.7432	9.17%
5	-2.6115	-4.1173	9.99%
6	-2.3492	-4.1039	-0.33%

The measurement errors are i.i.d. zero-mean Gaussian with standard deviation *σ *= 0.3.

In Table 3, we summarize the estimated values of *D *obtained from seven different perturbation experiments (logarithmic sampling is used with *Q *= 6). Moreover, we report the *D *values obtained by repeating an experiment that does not use molecular perturbations. Experimental replication may be an effective approach to obtain additional data, especially when molecular perturbations are costly or difficult to apply. Our formulation allows us to consider this scenario by setting *π_p _*= 0, for every *p *∈ P. The data collected this way correspond to repeating the same experiment *P *+ 1 times, where *P *is the number of elements in P. The maximum experimental replication considered in Table 3 uses *P *= 3, which corresponds to repeating the same experiment four times. This produces the same amount of data as the data obtained by perturbing the initial concentrations of Shc*, Grb2, and Sos, one at a time. The values depicted in Table 3 suggest that perturbing the initial concentrations of Shc*, Grb2, and Sos may be the right thing to do, since this produces the lowest value of *D *and, thus, it may result in better estimation performance as compared to perturbing the initial concentrations of one or two of these molecular species. In this case, however, it may also be acceptable to replicate an experiment that does not use molecular perturbations, since the minimum value of *D *is only 9.31% lower than the *D *value obtained by repeating the experiment four times.

**Table 3:** Estimated values of the *D*-optimal criterion for different replications and perturbations.

Perturbation	*D*
NO: 1 replication	-3.0123
NO: 2 replications	-3.4950
NO: 3 replications	-3.7544

YES: Shc*	-3.1398
YES: Grb2	-3.0747
YES: Sos	-3.4531
YES: Shc*, Grb2	-3.9279
YES: Shc*, Sos	-3.7716
YES: Grb2, Sos	-3.6363
YES: Shc*, Grb2, Sos	-4.1039

The measurement errors are i.i.d. zero-mean Gaussian with standard deviation *σ *= 0.3.

Logarithmic sampling is used with *Q *= 6.

**Figure 3:**
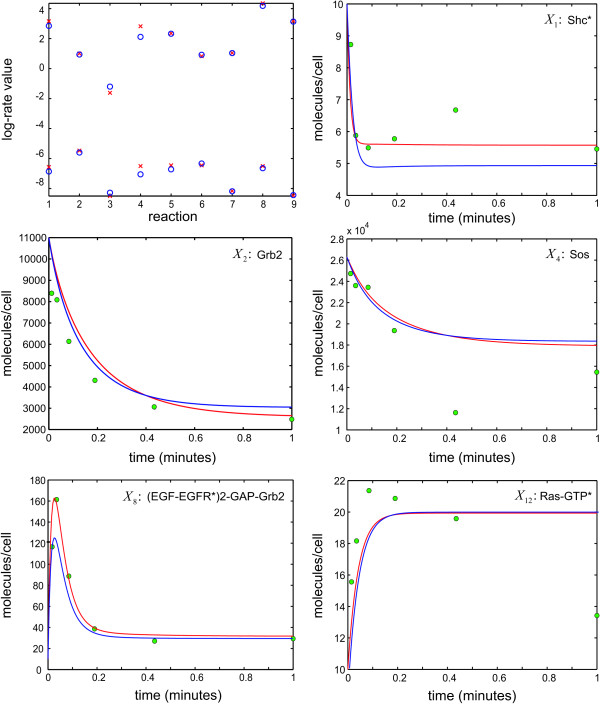
**True (red) vs. estimated (blue) log-rate values and selected molecular dynamics in the unperturbed biochemical reaction system depicted in Figure 1**. The results are based on measuring the dynamics in the unperturbed and perturbed systems at *Q *= 6 logarithmically-spaced time points (green circles). Perturbations are applied on the initial concentrations of Shc*, Grb2, and Sos, one at a time. The measurement errors are correlated zero-mean Gaussian with standard deviation *σ *= 0.3.

One of the underlying assumptions associated with the proposed Bayesian analysis algorithm is that the measurement errors are statistically independent, following a zero-mean Gaussian distribution with standard deviation *σ*. To assess the adequacy of this assumption and evaluate its implication on estimation performance, we depict in Table 4 calculated median and maximum absolute error values obtained when the measurement errors $\eta_{n}^{\left(p\right)}$ in (5) are i.i.d. zero-mean Gaussian with standard deviation *σ*, i.i.d. zero-mean uniform within the interval $\left[-\sqrt{3}\sigma ,\sqrt{3}\sigma \right]$, with standard deviation *σ*, and correlated zero-mean stationary Gaussian with autocorrelation $\text{E}[\eta_{n}^{\left(p\right)}(t_{1})\eta_{n}^{\left(p\right)}(t_{2})]=\sigma^{2}\exp\{-|t_{1}-t_{2}|\}$. We consider different values for the standard deviation, namely *σ *= 0.1, 0.2, 0.3, 0.4, 0.5, and measure the concentration dynamics in the unperturbed and perturbed systems at *Q *= 6 logarithmically spaced time points. Table 4 shows clearly that violation of the i.i.d. Gaussian assumption may lead to reduction in estimation accuracy, especially when the measurement errors are correlated, due to an increase in the maximum absolute error values. However, the calculated median absolute error values indicate that the proposed algorithm is relatively robust to the statistical behavior of the measurement errors, producing reasonable estimates for at least half of the concentration dynamics. In Figure 3, we depict results obtained by the proposed Bayesian analysis algorithm when measuring the concentration dynamics in the unperturbed and perturbed systems at *Q *= 6 logarithmically-spaced time points (green circles), with the measurements being corrupted by correlated zero-mean stationary Gaussian errors with standard deviation *σ *= 0.3. These results compare favorably to the ones depicted in Figure 2. In this case, the calculated median absolute error value is 1.48 × 10^-2^, which is 62.8% smaller that the value obtained when the errors are i.i.d. zero-mean Gaussian, whereas, the calculated maximum absolute error value is 7.32 × 10^-2^, which is 31.7% larger that the value obtained when the errors are i.i.d. zero-mean Gaussian.

**Table 4:** Median and maximum absolute error values under a variety of measurement error conditions.

mean = 0	i.i.d. Gaussian	i.i.d. Uniform	correlated Gaussian
*σ *= 0.1	3.98 × 10^-3^	8.64 × 10^-3^	1.48 × 10^-2^
	5.56 × 10^-2^	4.81 × 10^-2^	7.32 × 10^-2^
*σ *= 0.2	1.01 × 10^-2^	1.78 × 10^-2^	3.09 × 10^-2^
	8.29 × 10^-2^	1.30 × 10^-1^	1.89 × 10^-1^
*σ *= 0.3	3.03 × 10^-2^	1.78 × 10^-2^	3.05 × 10^-2^
	1.68 × 10^-1^	1.30 × 10^-1^	2.46 × 10^-1^
*σ *= 0.4	2.19 × 10^-2^	2.56 × 10^-2^	1.04 × 10^-1^
	2.27 × 10^-1^	1.41 × 10^-1^	3.67 × 10^-1^
*σ *= 0.5	2.67 × 10^-2^	3.86 × 10^-2^	6.43 × 10^-2^
	2.48 × 10^-1^	3.32 × 10^-1^	3.10 × 10^-1^

Logarithmic sampling is used with *Q *= 6.

## Conclusions

In this paper, we have introduced a novel Bayesian analysis technique for estimating the kinetic parameters (rate constants) of a closed biochemical reaction system from time measurements of noisy concentration dynamics. The proposed procedure enjoys a clear advantage over other published estimation techniques: the estimated kinetic parameters satisfy the Wegscheider conditions imposed by the fundamental laws of thermodynamics. As a consequence, it always leads to physically plausible biochemical reaction systems.

From a statistical perspective, there are additional advantages for thermodynamically restricting the kinetic parameters of a biochemical reaction system to satisfy the Wegscheider conditions. This may be seen through the well-known bias-variance tradeoff in estimation [27]. The mean squared error of a given estimator can be decomposed into a bias term and a variance term. In general, imposing constraints on the estimator may increase its bias but decrease its variance (hence the tradeoff). However, if the true parameter values satisfy the constraints, then the variance may decrease without increasing the bias term [27]. Since the true values of the kinetic parameters must lie on the thermodynamically feasible manifold in the parameter space, confining the Bayesian estimator to this manifold (which is of lower dimension than the parameter space itself) may lead to lower mean squared error due to a smaller variance. Since the thermodynamically feasible manifold is of lower dimension than the parameter space, gains in variance (and hence improvements in the mean squared error) are expected to be large. This may be seen through the "curse of dimensionality," which refers to the exponential increase in the volume of the parameter space as its dimension grows, making estimation exponentially harder in higher dimensional spaces (in our example, the unconstrained parameter space has 12.5% more dimensions than the thermodynamically feasible subspace). The Wegscheider conditions reduce the dimensionality of the parameter space to a feasible region in which estimation may be easier. Thus, the proposed Bayesian analysis procedure improves on other estimation techniques by producing a statistically superior, physically meaningful and plausible estimate for the kinetic parameters of a closed biochemical reaction system.

The Bayesian analysis methodology discussed in this paper has been formulated by assuming that all initial concentrations and perturbations are precisely known and that concentration measurements can be obtained by directly sampling all system dynamics. However, current experimental practices in quantitative systems biology restrict the amount and type of data that can be collected from living cells. As a consequence, further research is needed to develop approaches that can accommodate this important issue and make a Bayesian analysis approach to parameter estimation better applicable to systems biology problems.

If the initial concentrations and the perturbations applied on these concentrations are not known, then we may try to estimate them together with the unknown kinetic parameters. Although formulation of this problem is similar to the one considered in this paper, the additional computational burden will be substantial. Moreover, while quantitative biochemical techniques are improving, the vast majority of data available in problems of systems biology are obtained by measuring ratios of molecular concentrations (e.g., by using techniques such as SILAC [37]). Estimation of the rate constants of a biochemical reaction system from concentration measurements available as ratios relative to a reference system requires special consideration and extensive modification of the proposed Bayesian analysis procedure. Finally, it is very important to address the problem of missing observations. This is a common problem in systems biology, since it is not possible to monitor and measure the concentrations of all molecular species present in the system. Although appropriate modifications to the proposed algorithm can lead to a Bayesian analysis approach that can handle missing data, we think that development of a practically effective way to address this problem is challenging. Our future plan is to expand and improve the Bayesian analysis procedure discussed in this paper in order to provide practical solutions to the previous problems.

It is worth noting here that the estimation procedure suggested in this paper applies only to closed biochemical reaction systems (or to approximations of closed systems embedded in a larger open system). However, a cell is an open system, since it effectively interacts with its environment. If we include the cell's environment into our system and monitor the combined system until steady-state (i.e., until cell death), then we would have the necessary closed system. Unfortunately, this is clearly an unrealistic scenario. As a consequence, there is also a need to develop a theoretical and computational approach for dealing with thermodynamically consistent parameter estimation in open biochemical reaction systems.

To conclude, it has been argued in a recent paper [33] that most models of computational systems biology are "sloppy," in the sense that many parameters of such models do not appreciably alter system behavior. A key conclusion of this paper is that collective fitting procedures (such as the Bayesian analysis technique presented in the present paper) are far more desirable than piecewise construction of a biochemical reaction system model from individual parameter estimates (which is how most models are constructed when investigators scour the literature for individual rate constant values). Moreover, it has been pointed out in [33] that using a method to obtain precise parameter values may be difficult, even with an unlimited amount of data, since the behavior of a sloppy model is insensitive to the values of most parameters. As a consequence, the authors suggest that, instead of focusing on the quality of parameter estimation, it will be more wise to focus on the quality of prediction achieved by an estimated model (as we have also argued in this paper).

To a certain extent, our Bayesian analysis approach addresses some of the issues raised in [33]. By imposing the Wegscheider conditions on the kinetic parameters of a biochemical reaction system, we can effectively constrain these parameters to a thermodynamically feasible manifold in the parameter space, thus reducing sloppiness. Moreover, we can effectively use the RMSE values and the *D*-optimal criterion to determine an appropriate experimental design and distinguish those estimated values that can be trusted from those that cannot. For example, if the RMSE value associated with a kinetic parameter is small, then we may trust these values. On the other hand, a large RMSE value may indicate high uncertainty in the estimated parameter values, which may be untrustworthy. As we mentioned before, if a sensitivity analysis approach, such as the one proposed in [38], indicates that the kinetic parameters associated with large RMSE values are influential parameters, then we must reduce these RMSE values to an acceptable level of uncertainty by adopting a new and more effective experimental design approach. On the other hand, if these parameters correspond to a non-influential reaction, then we can accept the estimated values with no further consideration, since high uncertainty in the exact values of these parameters will not affect the predicted concentration dynamics.

## Authors' contributions

JG developed the basic Bayesian analysis framework, derived most theoretical results, coded a substantial portion of the software, and wrote the final version of the paper. GJ derived a number of theoretical results, generated much of the material in Additional files 2 &3, coded much of the final version of the software, and interpreted the obtained computational results. XZ and JG advised on several theoretical aspects of the paper, algorithm design, and data analysis, and wrote early versions of the software. GJ and JG advised on various theoretical aspects of the paper, on algorithm design, and data analysis. All authors read and approved the final version of the paper.

## Supplementary Material

Additional file 1 **In this document, we provide theoretical details necessary to understand the Bayesian analysis approach introduced in the Main text**. Click here for file

Additional file 2 **This document contains a detailed description of the computational algorithms used for implementing various steps of the proposed Bayesian analysis approach**. Click here for file

Additional file 3 **In this document, we list the biochemical reactions associated with our numerical example and provide thermodynamically consistent values for the rate constants as well as appropriate values for the initial molecular concentrations**. Click here for file
