# Supplementary material for: Thermodynamically consistent Bayesian analysis of closed biochemical reaction systems
Source: BMC Bioinformatics. 2010 Nov 5;11:547. doi: 10.1186/1471-2105-11-547 (PMC3248051; doi:10.1186/1471-2105-11-547)
Supplement: Additional file 1 — In this document, we provide theoretical details necessary to understand the Bayesian analysis approach introduced in the Main text. [file 1471-2105-11-547-S1.PDF]

ADDITIONAL FILE 1

Thermodynamically consistent Bayesian analysis of closed biochemical reaction systems

THEORY

Garrett Jenkinson,[1] Xiaogang Zhong,[2] and John Goutsias*[1]

[1]Whitaker Biomedical Engineering Institute, The Johns Hopkins University, Baltimore, MD 21218, USA
[2]Department of Applied Mathematics and Statistics, The Johns Hopkins University, Baltimore, MD 21218, USA

Email: Garrett Jenkinson - jenkinson@jhu.edu; Xiaogang Zhong - xzhong4@jhu.edu; John Goutsias*- goutsias@jhu.edu;

*Corresponding author

In this document, we provide theoretical details necessary to understand the Bayesian analysis approach introduced in the Main text. We start by deriving a general formula for the posterior density associated with our Bayesian analysis approach. We then discuss the Wegscheider conditions and their implications on the reaction rate constants of a closed biochemical reaction system. Subsequently, we derive appropriate prior probability density functions for the log-equilibrium constants as well as for the log-rate constants, and discuss a practical method for determining the hyperparameters associated with these priors. Finally, we present mathematical arguments to support our belief that the posterior mode should be a more preferable estimator of the kinetic parameters of a biochemical reaction system than the posterior mean.

Bayesian analysis

To develop a Bayesian analysis approach for estimating the kinetic parameters (rate constants) of a biochemical reaction system, we take the log-rate constants $\boldsymbol{\kappa}$ and the error variance σ^2 to be random variables. Note however that the probability density function of $\boldsymbol{\kappa}$ we consider in this paper depends on the log-equilibrium constants $\boldsymbol{z} = \{z_m, m \in \mathcal{M}\}$, where $z_m := \ln(k_{2m-1}/k_{2m})$, which we treat as *hyperparameters* (details to follow). Since we do not know the exact values of these hyperparameters, we take them to be random variables as well. By following this approach, we can write the *posterior* joint density of $\boldsymbol{\kappa}$, $\boldsymbol{z}$, and σ^2, given data $\boldsymbol{y}$, as[1]

$$p(\boldsymbol{\kappa}, \boldsymbol{z}, \sigma^2 \mid \boldsymbol{y}) = p(\sigma^2 \mid \boldsymbol{\kappa}, \boldsymbol{y}) p(\boldsymbol{\kappa} \mid \boldsymbol{z}, \boldsymbol{y}) p(\boldsymbol{z} \mid \boldsymbol{y}) \,. \tag{S-1.1}$$

Since our main objective is to estimate $\boldsymbol{\kappa}$, we are interested in evaluating the posterior density $p(\boldsymbol{\kappa} \mid \boldsymbol{y})$. To do so, we must integrate the log-equilibrium constants $\boldsymbol{z}$ and the error variance σ^2 out of the posterior joint density $p(\boldsymbol{\kappa}, \boldsymbol{z}, \sigma^2 \mid \boldsymbol{y})$. In this case,

$$p(\boldsymbol{\kappa} \mid \boldsymbol{y}) = \int \int p(\boldsymbol{\kappa}, \boldsymbol{z}, \sigma^2 \mid \boldsymbol{y}) d\sigma^2 d\boldsymbol{z} = \int p(\boldsymbol{\kappa} \mid \boldsymbol{z}, \boldsymbol{y}) p(\boldsymbol{z} \mid \boldsymbol{y}) d\boldsymbol{z}, \tag{S-1.2}$$

by virtue of (S-1.1). On the other hand,

$$p(\boldsymbol{\kappa} \mid \boldsymbol{z}, \boldsymbol{y}) = \frac{p(\boldsymbol{y} \mid \boldsymbol{\kappa}) p(\boldsymbol{\kappa} \mid \boldsymbol{z}) p(\boldsymbol{z})}{p(\boldsymbol{z} \mid \boldsymbol{y}) p(\boldsymbol{y})} \,. \tag{S-1.3}$$

As a consequence of (S-1.2) and (S-1.3), we have that

$$\begin{aligned} p(\boldsymbol{\kappa} \mid \boldsymbol{y}) &= \frac{p(\boldsymbol{y} \mid \boldsymbol{\kappa})}{p(\boldsymbol{y})} \int p(\boldsymbol{\kappa} \mid \boldsymbol{z}) p(\boldsymbol{z}) d\boldsymbol{z} \\ &\propto p(\boldsymbol{y} \mid \boldsymbol{\kappa}) \int p(\boldsymbol{\kappa} \mid \boldsymbol{z}) p(\boldsymbol{z}) d\boldsymbol{z}, \end{aligned} \tag{S-1.4}$$

where $p \propto q$ denotes that p is proportional to q, whereas,

$$p(\boldsymbol{y} \mid \boldsymbol{\kappa}) = \int p(\boldsymbol{y} \mid \boldsymbol{\kappa}, \sigma^2) p(\sigma^2 \mid \boldsymbol{\kappa}) d\sigma^2. \tag{S-1.5}$$

The term $p(\boldsymbol{y} \mid \boldsymbol{\kappa})$ is simply the average of the likelihood $p(\boldsymbol{y} \mid \boldsymbol{\kappa}, \sigma^2)$ over the conditional prior density $p(\sigma^2 \mid \boldsymbol{\kappa})$ of σ^2 given the values of the log-rate constants $\boldsymbol{\kappa}$. We refer to $p(\boldsymbol{y} \mid \boldsymbol{\kappa})$ as the "effective" likelihood. Moreover, we refer to $\int p(\boldsymbol{\kappa} \mid \boldsymbol{z}) p(\boldsymbol{z}) d\boldsymbol{z}$ as the "effective" prior, since this term contains the prior information about the log-rate constants $\boldsymbol{\kappa}$ after the prior information about the log-equilibrium constants is integrated out of the problem.

[1]It is clear that $\boldsymbol{z}$ can be directly calculated from $\boldsymbol{\kappa}$. Consequently, conditioning on both $\boldsymbol{\kappa}$ and $\boldsymbol{z}$ is equivalent to conditioning only on $\boldsymbol{\kappa}$. Therefore, $p(\sigma^2 \mid \boldsymbol{\kappa}, \boldsymbol{z}, \boldsymbol{y}) = p(\sigma^2 \mid \boldsymbol{\kappa}, \boldsymbol{y})$ and $p(\boldsymbol{y} \mid \boldsymbol{\kappa}, \boldsymbol{z}) = p(\boldsymbol{y} \mid \boldsymbol{\kappa})$.

Wegscheider conditions

As we mentioned in the Main text, the thermodynamic constraints given by Equation (10) are equivalent to the Wegscheider conditions given by Equation (11). To show this fact, let us first assume that Equation (10) is satisfied. Then, for every $\boldsymbol{r} = \{r_m, m \in \mathcal{M}\} \in \text{null}(\mathbb{S})$,

$$\begin{aligned}
\ln \prod_{m\in\mathcal{M}} \left(\frac{k_{2m-1}}{k_{2m}}\right)^{r_m} &= \sum_{m\in\mathcal{M}} r_m \ln \frac{k_{2m-1}}{k_{2m}} \\
&= \sum_{m\in\mathcal{M}} r_m \ln \frac{\prod_{n\in\mathcal{N}} [\overline{x}_n^{(p)}]^{\nu'_{nm}}}{\prod_{n\in\mathcal{N}} [\overline{x}_n^{(p)}]^{\nu_{nm}}} \\
&= \sum_{m\in\mathcal{M}} r_m \ln \prod_{n\in\mathcal{N}} [\overline{x}_n^{(p)}]^{s_{nm}} \\
&= \sum_{n\in\mathcal{N}} \left(\sum_{m\in\mathcal{M}} s_{nm} r_m\right) \ln \overline{x}_n^{(p)} \\
&= 0,
\end{aligned}$$

where $\{\overline{x}_n^{(p)}, n \in \mathcal{N}\}$ are the stationary concentrations in the system when the initial concentration of the pth molecular species is perturbed. This shows Equation (11).

On the other hand, if Equation (11) is satisfied, then

$$\begin{aligned}
\sum_{m\in\mathcal{M}} r_m \ln \frac{k_{2m-1}}{k_{2m}} \frac{\prod_{n\in\mathcal{N}} [\overline{x}_n^{(p)}]^{\nu_{nm}}}{\prod_{n\in\mathcal{N}} [\overline{x}_n^{(p)}]^{\nu'_{nm}}} &= \sum_{m\in\mathcal{M}} \ln \left(\frac{k_{2m-1}}{k_{2m}}\right)^{r_m} - \sum_{m\in\mathcal{M}} r_m \ln \prod_{n\in\mathcal{N}} [\overline{x}_n^{(p)}]^{s_{nm}} \\
&= \sum_{m\in\mathcal{M}} \ln \left(\frac{k_{2m-1}}{k_{2m}}\right)^{r_m} - \sum_{n\in\mathcal{N}} \left(\sum_{m\in\mathcal{M}} s_{nm} r_m\right) \ln \overline{x}_n^{(p)} \\
&= \sum_{m\in\mathcal{M}} \ln \left(\frac{k_{2m-1}}{k_{2m}}\right)^{r_m} = 0, \qquad \text{(S-1.6)}
\end{aligned}$$

for every $\boldsymbol{r} \in \text{null}(\mathbb{S})$. We can take the mth element r_m of $\boldsymbol{r}$ to be

$$r_m = k_{2m-1} \prod_{n\in\mathcal{N}} [\overline{x}_n^{(p)}]^{\nu_{nm}} - k_{2m} \prod_{n\in\mathcal{N}} [\overline{x}_n^{(p)}]^{\nu'_{nm}},$$

since, from Equations (2) and (4) in the Main text, we have $\mathbb{S}\boldsymbol{r} = 0$, which implies that $\boldsymbol{r} \in \text{null}(\mathbb{S})$. Note now that

$$\sum_{m\in\mathcal{M}} \left(k_{2m-1} \prod_{n\in\mathcal{N}} [\overline{x}_n^{(p)}]^{\nu_{nm}} - k_{2m} \prod_{n\in\mathcal{N}} [\overline{x}_n^{(p)}]^{\nu'_{nm}}\right) \ln \frac{k_{2m-1}}{k_{2m}} \frac{\prod_{n\in\mathcal{N}} [\overline{x}_n^{(p)}]^{\nu'_{nm}}}{\prod_{n\in\mathcal{N}} [\overline{x}_n^{(p)}]^{\nu_{nm}}} = 0,$$

by virtue of (S-1.6). However, each term in the previous sum is nonnegative. Hence, for the sum to be equal to zero, each term must be zero, which implies the steady-state thermodynamic constrains given by Equation (10) in the Main text.

We will now show that the Wegscheider conditions are satisfied for all $\boldsymbol{r} \in \text{null}(\mathbb{S})$ so long as they are satisfied for $M_2 = M - M_1$ basis vectors $\{\boldsymbol{r}(j), j = 1, 2, \ldots, M_1\}$ of the null space of $\mathbb{S}$, where $M_1 = \text{rank}(\mathbb{S})$. Note that, for any $\boldsymbol{r} \in \text{null}(\mathbb{S})$,

$$\boldsymbol{r} = \sum_{j=1}^{M_2} a_j \boldsymbol{r}(j),$$

for some scalar coefficients $\alpha_j, j = 1, 2, \ldots, M_2$. As a consequence, and from Equations (8) and (11) in the Main text, we have that

$$\begin{aligned}
\ln \prod_{m \in \mathcal{M}} \left(\frac{k_{2m-1}}{k_{2m}} \right)^{r_m} &= \sum_{m \in \mathcal{M}} r_m \ln \frac{k_{2m-1}}{k_{2m}} \\
&= \sum_{m \in \mathcal{M}} r_m z_m \\
&= \sum_{m \in \mathcal{M}} \left[\sum_{j=1}^{M_2} a_j r_m(j) \right] z_m \\
&= \sum_{j=1}^{M_2} a_j \sum_{m \in \mathcal{M}} z_m r_m(j) \\
&= \sum_{j=1}^{M_2} a_j \sum_{m \in \mathcal{M}} r_m(j) \ln \frac{k_{2m-1}}{k_{2m}} \\
&= \sum_{j=1}^{M_2} a_j \ln \prod_{m \in \mathcal{M}} \left(\frac{k_{2m-1}}{k_{2m}} \right)^{r_m(j)} \\
&= 0,
\end{aligned}$$

for every $\boldsymbol{r} = \{r_m, m \in \mathcal{M}\} \in \text{null}(\mathbb{S})$, since the Wegscheider conditions are assumed to be satisfied by the basis vectors of the null space of $\mathbb{S}$. This shows that the Wegscheider conditions are also satisfied for every $\boldsymbol{r} \in \text{null}(\mathbb{S})$.

Let us now rearrange the columns and rows of the stoichiometry matrix $\mathbb{S}$ (by appropriately relabeling the molecular species and reactions) so that the first M_1 columns are linearly independent, whereas, the

remaining M_2 columns are linearly dependent on the first columns. In this case, we can write the stoichiometry matrix $\mathbb{S}$ in the following block matrix form:

$$\mathbb{S} = \begin{bmatrix} \mathbb{S}_{11} & \mathbb{S}_{12} \\ \mathbb{S}_{21} & \mathbb{S}_{22} \end{bmatrix},$$

where $\mathbb{S}_{11}$ is an *invertible* $M_1 \times M_1$ matrix, whereas, $\mathbb{S}_{12}$, $\mathbb{S}_{21}$, and $\mathbb{S}_{22}$ are $M_1 \times M_2$, $(N - M_1) \times M_1$, and $(N - M_1) \times M_2$ matrices, respectively. It is a well-known fact (e.g., see [1]) that the general solution of $\mathbb{S}\boldsymbol{r} = \mathbf{0}$ is given by $\boldsymbol{r}' = -\mathbb{S}_{11}^{-1}\mathbb{S}_{12}\boldsymbol{r}''$, for an arbitrary $\boldsymbol{r}''$, where $\boldsymbol{r}'$, $\boldsymbol{r}''$ are $M_1 \times 1$ and $M_2 \times 1$ vectors, respectively, such that

$$\boldsymbol{r} = \begin{bmatrix} \boldsymbol{r}' \\ \boldsymbol{r}'' \end{bmatrix}.$$

This implies that the columns of matrix

$$\mathbb{B} = \begin{bmatrix} -\mathbb{S}_{11}^{-1}\mathbb{S}_{12} \\ \mathbb{I}_{M_2} \end{bmatrix},$$

where $\mathbb{I}_{M_2}$ is the $M_2 \times M_2$ identity matrix, form a basis for the null space of $\mathbb{S}$. As a consequence of this result and the fact that the Wegscheider conditions are satisfied so long they are satisfied by a basis vector of null($\mathbb{S}$), we can conclude that the Wegscheider conditions, given by Equation (11) in the Main text, are equivalent to the following conditions [2]:

$$\kappa_{2m'} = \kappa_{2m'-1} + \sum_{m \in \mathcal{M}_1} [\mathbb{S}_{11}^{-1}\mathbb{S}_{12}]_{m,m'} (\kappa_{2m} - \kappa_{2m-1}), \quad \text{for every } m' \in \mathcal{M}_2, \tag{S-1.7}$$

where $\mathcal{M}_1 = \{1, 2, \ldots, M_1\}$, $\mathcal{M}_2 = \{M_1 + 1, M_1 + 2, \ldots, M\}$, and $[\mathbb{S}_{11}^{-1}\mathbb{S}_{12}]_{m,m'}$ is the element of the mth row and the m'^{th} column of matrix $\mathbb{S}_{11}^{-1}\mathbb{S}_{12}$.

Equation (S-1.7) allows us to specify arbitrary values for the forward and reverse log-rate constants $\{\kappa_{2m-1}, m \in \mathcal{M}\}$, $\{\kappa_{2m}, m \in \mathcal{M}_1\}$, and calculate the reverse log-rate constants $\{\kappa_{2m}, m \in \mathcal{M}_2\}$ so that the Wegscheider conditions are satisfied. If we denote by $\boldsymbol{\kappa}_f$ the $(M + M_1)$ "free" log-rate constants $\{\kappa_{2m-1}, m \in \mathcal{M}, \kappa_{2m}, m \in \mathcal{M}_1\}$ and by $\boldsymbol{\kappa}_d$ the M_2 "dependent" log-rate constants $\{\kappa_{2m}, m \in \mathcal{M}_2\}$, then we can write (S-1.7) in the following compact form:

$$\boldsymbol{\kappa}_d = \mathbb{W}\boldsymbol{\kappa}_f, \tag{S-1.8}$$

where $\mathbb{W}$ is the $(M - M_1) \times (M + M_1)$ matrix implementing the right hand side of (S-1.7), given by

$$\mathbb{W} = \left[-(\mathbb{S}_{11}^{-1}\mathbb{S}_{12})^T \mid \mathbb{I}_{M_2} \mid (\mathbb{S}_{11}^{-1}\mathbb{S}_{12})^T \right]. \tag{S-1.9}$$

Thus, for any arbitrary $\boldsymbol{\kappa}_f$, we can construct a thermodynamically feasible set of kinetic parameters by determining the dependent parameters $\boldsymbol{\kappa}_d$ according to (S-1.8), and by setting $\boldsymbol{\kappa} = \{\boldsymbol{\kappa}_f, \boldsymbol{\kappa}_d\}$.

Prior density of log-equilibrium constants

From Equations (8) and (10) in the Main text, we have that

$$z_m = \sum_{n \in \mathcal{N}} s_{nm} \ln \overline{x}_n^{(p)}, \quad \text{for all } \; p \in \mathcal{P},$$

where $s_{nm} = \nu'_{nm} - \nu_{nm}$ is the net stoichiometry coefficient and $\{\overline{x}_n^{(p)}, n \in \mathcal{N}\}$ are the stationary concentrations when the initial concentration of the pth molecular species is perturbed. Therefore,

$$z_m = \frac{1}{P+1} \sum_{p \in \mathcal{P}} \sum_{n \in \mathcal{N}} s_{nm} \ln \overline{x}_n^{(p)}, \quad \text{for all } \; m \in \mathcal{M}. \tag{S-1.10}$$

On the other hand, Equation (5) in the Main text gives

$$\sum_{p \in \mathcal{P}} \sum_{n \in \mathcal{N}} s_{nm} \ln x_n^{(p)}(t_q) = \sum_{p \in \mathcal{P}} \sum_{n \in \mathcal{N}} s_{nm} y_n^{(p)}(t_q) - \sum_{p \in \mathcal{P}} \sum_{n \in \mathcal{N}} s_{nm} \eta_n^{(p)}(t_q). \tag{S-1.11}$$

If we assume that the biochemical reaction system and all its perturbed versions are sufficiently close to steady-state at some time point t_{Q+1}, then (S-1.10) and (S-1.11) approximately imply that

$$z_m = \widetilde{y}_m - \widetilde{\eta}_m, \tag{S-1.12}$$

where

$$\widetilde{y}_m := \frac{1}{P+1} \sum_{p \in \mathcal{P}} \sum_{n \in \mathcal{N}} s_{nm} y_n^{(p)}(t_{Q+1}), \tag{S-1.13}$$

and

$$\widetilde{\eta}_m := \frac{1}{P+1} \sum_{p \in \mathcal{P}} \sum_{n \in \mathcal{N}} s_{nm} \eta_n^{(p)}(t_{Q+1}).$$

To proceed, note that

$$\begin{aligned} p(\boldsymbol{z} \mid \overline{\boldsymbol{y}}) &= p(\boldsymbol{z} \mid \widetilde{\boldsymbol{y}}, \overline{\boldsymbol{y}}) \\ &= \int p(\boldsymbol{z}, \sigma^2 \mid \widetilde{\boldsymbol{y}}, \overline{\boldsymbol{y}}) d\sigma^2 \\ &= \int p(\boldsymbol{z} \mid \sigma^2, \widetilde{\boldsymbol{y}}, \overline{\boldsymbol{y}}) p(\sigma^2 \mid \widetilde{\boldsymbol{y}}, \overline{\boldsymbol{y}}) d\sigma^2 \\ &= \int p(\boldsymbol{z} \mid \sigma^2, \widetilde{\boldsymbol{y}}) p(\sigma^2 \mid \overline{\boldsymbol{y}}) d\sigma^2. \end{aligned} \tag{S-1.14}$$

This is due to the fact that $\widetilde{\boldsymbol{y}} := \{\widetilde{y}_m, m \in \mathcal{M}\}$ can be calculated from $\overline{\boldsymbol{y}} := \{y_n^{(p)}(t_{Q+1}), n \in \mathcal{N}, p \in \mathcal{P}\}$ by means of (S-1.13), in which case $p(\boldsymbol{z} \mid \widetilde{\boldsymbol{y}}, \overline{\boldsymbol{y}}) = p(\boldsymbol{z} \mid \overline{\boldsymbol{y}})$ and $p(\sigma^2 \mid \widetilde{\boldsymbol{y}}, \overline{\boldsymbol{y}}) = p(\sigma^2 \mid \overline{\boldsymbol{y}})$. Moreover, since

$z_m = \widetilde{y}_m - \widetilde{\eta}_m$, $\overline{\boldsymbol{y}}$ does not provide further information about $\boldsymbol{z}$, given σ^2 and $\widetilde{\boldsymbol{y}}$, in which case $p(\boldsymbol{z} \mid \sigma^2, \widetilde{\boldsymbol{y}}, \overline{\boldsymbol{y}}) = p(\boldsymbol{z} \mid \sigma^2, \widetilde{\boldsymbol{y}})$.

Equation (S-1.14) implies that, in order to calculate the probability density function $p(\boldsymbol{z} \mid \overline{\boldsymbol{y}})$, we must determine the probability density functions $p(\boldsymbol{z} \mid \sigma^2, \widetilde{\boldsymbol{y}})$ and $p(\sigma^2 \mid \overline{\boldsymbol{y}})$. Note that, given σ^2 and $\widetilde{\boldsymbol{y}}$, the log-equilibrium constants $\boldsymbol{z}$ follow a multivariate Gaussian distribution with means and covariances

$$\mathrm{E}[z_m \mid \sigma^2, \widetilde{\boldsymbol{y}}] = \widetilde{y}_m \qquad \text{and} \qquad \mathrm{cov}[z_m, z_{m'} \mid \sigma^2, \widetilde{\boldsymbol{y}}] = \frac{\sigma^2}{P+1} \sum_{n \in \mathcal{N}} s_{nm} s_{nm'} \,.$$

This implies that

$$p(\boldsymbol{z} \mid \sigma^2, \widetilde{\boldsymbol{y}}) = \frac{(P+1)^{M/2}}{(2\pi)^{M/2} \sigma^M |\mathbb{H}|^{1/2}} \exp\left\{ -\frac{P+1}{2\sigma^2} (\boldsymbol{z} - \widetilde{\boldsymbol{y}})^T \mathbb{H}^{-1} (\boldsymbol{z} - \widetilde{\boldsymbol{y}}) \right\}, \tag{S-1.15}$$

where $\mathbb{H}$ is an $M \times M$ matrix with elements $h_{mm'} = \sum_{n \in \mathcal{N}} s_{nm} s_{nm'}$. Note that $\mathbb{H} = \mathbb{S}^T \mathbb{S}$, where $\mathbb{S}$ is the $N \times M$ stoichiometry matrix of the biochemical reaction system with elements s_{nm}.

To determine the probability density function $p(\sigma^2 \mid \overline{\boldsymbol{y}})$ of the error variance, note that

$$p(\overline{\boldsymbol{x}}, \sigma^2 \mid \overline{\boldsymbol{y}}) = \frac{p(\overline{\boldsymbol{y}} \mid \overline{\boldsymbol{x}}, \sigma^2) p(\overline{\boldsymbol{x}}, \sigma^2)}{p(\overline{\boldsymbol{y}})}, \tag{S-1.16}$$

where $\overline{\boldsymbol{x}} := \{\ln \overline{x}_n^{(p)}, n \in \mathcal{N}, p \in \mathcal{P}\}$ are the stationary concentrations when the initial concentration of the pth molecular species is perturbed. Moreover,

$$p(\overline{\boldsymbol{x}}, \sigma^2 \mid \overline{\boldsymbol{y}}) = p(\overline{\boldsymbol{x}} \mid \sigma^2, \overline{\boldsymbol{y}}) p(\sigma^2 \mid \overline{\boldsymbol{y}}). \tag{S-1.17}$$

As a consequence of (S-1.16) and (S-1.17), we have

$$\begin{aligned} p(\sigma^2 \mid \overline{\boldsymbol{y}}) &= \frac{p(\overline{\boldsymbol{x}}, \sigma^2 \mid \overline{\boldsymbol{y}})}{p(\overline{\boldsymbol{x}} \mid \sigma^2, \overline{\boldsymbol{y}})} \\ &= \frac{p(\overline{\boldsymbol{y}} \mid \overline{\boldsymbol{x}}, \sigma^2) p(\overline{\boldsymbol{x}}, \sigma^2)}{p(\overline{\boldsymbol{x}} \mid \sigma^2, \overline{\boldsymbol{y}}) p(\overline{\boldsymbol{y}})} \\ &= \frac{p(\overline{\boldsymbol{y}} \mid \overline{\boldsymbol{x}}, \sigma^2) p(\overline{\boldsymbol{x}}) p(\sigma^2)}{p(\overline{\boldsymbol{x}} \mid \sigma^2, \overline{\boldsymbol{y}}) p(\overline{\boldsymbol{y}})} \\ &\propto \frac{p(\overline{\boldsymbol{y}} \mid \overline{\boldsymbol{x}}, \sigma^2)}{p(\overline{\boldsymbol{x}} \mid \sigma^2, \overline{\boldsymbol{y}})} \, p(\sigma^2) \,, \end{aligned}$$

where we use the fact that $\overline{\boldsymbol{x}}$ and σ^2 are statistically independent (since $\overline{\boldsymbol{x}}$ is determined from $\boldsymbol{\kappa}$ and we have assumed in the Main text that $\boldsymbol{\kappa}$ and σ^2 are statistically independent). It is now not difficult to see that

$p(\overline{\boldsymbol{y}} \mid \overline{\boldsymbol{x}}, \sigma^2) = p(\overline{\boldsymbol{x}} \mid \sigma^2, \overline{\boldsymbol{y}})$, due to the statistical independence and Gaussianity of the error terms $\eta_n^{(p)}$ in Equation (5) of the Main text. Therefore,

$$p(\sigma^2 \mid \overline{\boldsymbol{y}}) = p(\sigma^2) = \frac{b^a}{\Gamma(a)} (\sigma^2)^{-(a+1)} e^{-b/\sigma^2}, \tag{S-1.18}$$

which does not depend on $\overline{\boldsymbol{y}}$, by virtue of Equation (9) in the Main text.

As a consequence of (S-1.14) and (S-1.18), we now have that

$$p(\boldsymbol{z} \mid \overline{\boldsymbol{y}}) = p(\boldsymbol{z} \mid \widetilde{\boldsymbol{y}}, \overline{\boldsymbol{y}}) = p(\boldsymbol{z} \mid \widetilde{\boldsymbol{y}}). \tag{S-1.19}$$

Finally, from (S-1.14), (S-1.15), (S-1.18), and (S-1.19), and after some straightforward, albeit tedious, algebraic manipulations, we can show that

$$\begin{aligned} p(\boldsymbol{z} \mid \widetilde{\boldsymbol{y}}) &\propto \int \frac{1}{\sigma^{2(a+1)+M}} \exp\left\{-\frac{P+1}{2\sigma^2}(\boldsymbol{z} - \widetilde{\boldsymbol{y}})^T \mathbb{H}^{-1} (\boldsymbol{z} - \widetilde{\boldsymbol{y}}) - \frac{b}{\sigma^2}\right\} d\sigma^2 \\ &\propto \left[\frac{2b}{P+1} + (\boldsymbol{z} - \widetilde{\boldsymbol{y}})^T \mathbb{H}^{-1} (\boldsymbol{z} - \widetilde{\boldsymbol{y}})\right]^{-(M/2+a)}. \end{aligned} \tag{S-1.20}$$

A problem associated with the previous formulation is that it may not be possible to evaluate the prior density $p(\boldsymbol{z} \mid \widetilde{\boldsymbol{y}})$ of the log-equilibrium constants, given by (S-1.20), since the matrix $\mathbb{H}$ may not be invertible. Indeed, if $\boldsymbol{r}$ is a (nonzero) vector in the null space of the $N \times M$ stoichiometry matrix $\mathbb{S}$ (i.e., if $\mathbb{S}\,\boldsymbol{r} = 0$), then $\boldsymbol{r}^T \mathbb{H}\, \boldsymbol{r} = \boldsymbol{r}^T \mathbb{S}^T \mathbb{S}\, \boldsymbol{r} = 0$, which shows that matrix $\mathbb{H}$ is positive semi-definite and, hence, not necessarily invertible.

To address the previous problem, we will follow a well-known technique known as *decorrelation* or *whitening* that allows us to transform the dependent random variables $\boldsymbol{z}$ into the statistically independent zero-mean random variables $\boldsymbol{z}_0$ and obtain a form of matrix $\mathbb{H}$ that is always invertible. Subsequently, this will allow us to derive a form for the probability density function $p(\boldsymbol{z} \mid \widetilde{\boldsymbol{y}})$ that we can always evaluate.

Let us consider an $(M - M_0)$-dimensional zero-mean Gaussian random vector $\boldsymbol{z}_0$ with identity covariance matrix, where M_0 in the number of zero eigenvalues of $\mathbb{H}$. Our first objective is to determine an $M \times (M - M_0)$ matrix $\mathbb{H}_0$, such that

$$\boldsymbol{z} = \mathbb{H}_0 \boldsymbol{z}_0 + \widetilde{\boldsymbol{y}}\,. \tag{S-1.21}$$

Note that $\mathrm{E}[\boldsymbol{z} \mid \sigma^2, \widetilde{\boldsymbol{y}}] = \widetilde{\boldsymbol{y}}$, as expected from (S-1.12), whereas, $\mathbb{H} = [(P+1)/\sigma^2]\, \mathbb{H}_0\, \mathbb{H}_0^T$. We can use singular value decomposition (SVD) [3] to decompose matrix $\mathbb{H} = \mathbb{S}^T \mathbb{S}$ into $\mathbb{H} = \mathbb{U}\mathbb{D}\mathbb{U}^T$, form the

$(M - M_0) \times (M - M_0)$ diagonal matrix $\mathbb{D}_0$ by removing the last M_0 zero singular values from $\mathbb{D}$ and the $M \times (M - M_0)$ submatrix $\mathbb{U}_0$ of $\mathbb{U}$ by removing the last M_0 columns of $\mathbb{U}$. Then, $\mathbb{H} = \mathbb{U}_0\mathbb{D}_0\mathbb{U}_0^T$, which implies $\mathbb{H}_0 = \sigma\, \mathbb{U}_0\mathbb{D}_0^{1/2}/\sqrt{P+1}$. Note now that $\mathbb{U}_0^T\mathbb{U}_0 = \mathbb{I}$, where $\mathbb{I}$ denotes the identity matrix. As a consequence, given vectors $\boldsymbol{z}$ and $\widetilde{\boldsymbol{y}}$, (S-1.21) has a unique solution, given by

$$\boldsymbol{z}_0 = \frac{\sqrt{P+1}}{\sigma}\, \mathbb{D}_0^{-1/2}\mathbb{U}_0^T(\boldsymbol{z} - \widetilde{\boldsymbol{y}})\,. \tag{S-1.22}$$

This formula transforms the dependent random variables $\boldsymbol{z}$ into the statistically independent zero-mean random variables $\boldsymbol{z}_0$.

Note now that

$$\begin{aligned}
p(\boldsymbol{z} \mid \sigma^2, \widetilde{\boldsymbol{y}}) &= \int p(\boldsymbol{z}, \boldsymbol{z}_0 \mid \sigma^2, \widetilde{\boldsymbol{y}})\, d\boldsymbol{z}_0 \\
&= \int p(\boldsymbol{z} \mid \boldsymbol{z}_0, \sigma^2, \widetilde{\boldsymbol{y}}) p(\boldsymbol{z}_0)\, d\boldsymbol{z}_0 \\
&= \frac{1}{(2\pi)^{(M-M_0)/2}} \int \delta(\boldsymbol{z} - \mathbb{H}_0\boldsymbol{z}_0 - \widetilde{\boldsymbol{y}}) \exp\left\{-\frac{1}{2}\, \boldsymbol{z}_0^T\boldsymbol{z}_0\right\} d\boldsymbol{z}_0 \\
&= \frac{\int \delta(\boldsymbol{z} - \mathbb{H}_0\boldsymbol{z}_0 - \widetilde{\boldsymbol{y}}) d\boldsymbol{z}_0}{(2\pi)^{(M-M_0)/2}} \exp\left\{-\frac{P+1}{2\sigma^2}(\boldsymbol{z} - \widetilde{\boldsymbol{y}})^T\mathbb{U}_0\mathbb{D}_0^{-1}\mathbb{U}_0^T(\boldsymbol{z} - \widetilde{\boldsymbol{y}})\right\} \\
&= \frac{\int \delta(\mathbb{H}_0\boldsymbol{z}_0)\, d\boldsymbol{z}_0}{(2\pi)^{(M-M_0)/2}} \exp\left\{-\frac{P+1}{2\sigma^2}(\boldsymbol{z} - \widetilde{\boldsymbol{y}})^T\mathbb{U}_0\mathbb{D}_0^{-1}\mathbb{U}_0^T(\boldsymbol{z} - \widetilde{\boldsymbol{y}})\right\},
\end{aligned}$$

by virtue of (S-1.21) and (S-1.22), where $\delta(\cdot)$ is the Dirac delta function. This result shows that

$$p(\boldsymbol{z} \mid \sigma^2, \widetilde{\boldsymbol{y}}) \propto \exp\left\{-\frac{P+1}{2\sigma^2}(\boldsymbol{z} - \widetilde{\boldsymbol{y}})^T\mathbb{U}_0\mathbb{D}_0^{-1}\mathbb{U}_0^T(\boldsymbol{z} - \widetilde{\boldsymbol{y}})\right\},$$

which leads to [compare with (S-1.20)]:

$$\begin{aligned}
p(\boldsymbol{z} \mid \widetilde{\boldsymbol{y}}) &\propto \int \frac{1}{\sigma^{2(a+1)}} \exp\left\{-\frac{P+1}{2\sigma^2}(\boldsymbol{z} - \widetilde{\boldsymbol{y}})^T\mathbb{U}_0\mathbb{D}_0^{-1}\mathbb{U}_0^T(\boldsymbol{z} - \widetilde{\boldsymbol{y}}) - \frac{b}{\sigma^2}\right\} d\sigma^2 \\
&\propto \left[\frac{2b}{P+1} + (\boldsymbol{z} - \widetilde{\boldsymbol{y}})^T\mathbb{U}_0\mathbb{D}_0^{-1}\mathbb{U}_0^T(\boldsymbol{z} - \widetilde{\boldsymbol{y}})\right]^{-a}.
\end{aligned}$$

Note that we can always evaluate this form of $p(\boldsymbol{z} \mid \widetilde{\boldsymbol{y}})$, since the diagonal matrix $\mathbb{D}_0$ is invertible.

If we now replace $p(\boldsymbol{z})$ by $p(\boldsymbol{z} \mid \widetilde{\boldsymbol{y}})$ in Equation (6) of the Main text, we have

$$\begin{aligned}
\int p(\boldsymbol{\kappa} \mid \boldsymbol{y}) d\boldsymbol{\kappa} &= \frac{1}{p(\boldsymbol{y})} \int\int p(\boldsymbol{y} \mid \boldsymbol{\kappa}) p(\boldsymbol{\kappa} \mid \boldsymbol{z}) p(\boldsymbol{z} \mid \widetilde{\boldsymbol{y}}) d\boldsymbol{\kappa} d\boldsymbol{z} \\
&= \frac{1}{p(\boldsymbol{y})} \int\int p(\boldsymbol{y} \mid \boldsymbol{\kappa}, \boldsymbol{z}) p(\boldsymbol{\kappa} \mid \boldsymbol{z}) p(\boldsymbol{z} \mid \widetilde{\boldsymbol{y}}) d\boldsymbol{\kappa} d\boldsymbol{z} \\
&= \frac{1}{p(\boldsymbol{y})} \int p(\boldsymbol{y} \mid \boldsymbol{z}) p(\boldsymbol{z} \mid \widetilde{\boldsymbol{y}}) d\boldsymbol{z} \\
&= \frac{1}{p(\boldsymbol{y})} \int p(\boldsymbol{y} \mid \boldsymbol{z}, \widetilde{\boldsymbol{y}}) p(\boldsymbol{z} \mid \widetilde{\boldsymbol{y}}) d\boldsymbol{z} \\
&= 1, \quad \text{for all } \boldsymbol{y},
\end{aligned}$$

since $p(\boldsymbol{y} \mid \boldsymbol{\kappa}, \boldsymbol{z}) = p(\boldsymbol{y} \mid \boldsymbol{\kappa})$ (see footnote 1), provided that $\widetilde{\boldsymbol{y}}$ is statistically independent of $\boldsymbol{y}$, in which case $p(\boldsymbol{y} \mid \boldsymbol{z}, \widetilde{\boldsymbol{y}}) = p(\boldsymbol{y} \mid \boldsymbol{z})$ and $p(\boldsymbol{y} \mid \widetilde{\boldsymbol{y}}) = p(\boldsymbol{y})$. Clearly, the independence between $\widetilde{\boldsymbol{y}}$ and $\boldsymbol{y}$ is a sufficient condition for the posterior density $p(\boldsymbol{\kappa} \mid \boldsymbol{y})$ to be proper [i.e., for $p(\boldsymbol{\kappa} \mid \boldsymbol{y})$ to be finite for every $\boldsymbol{y}$].

Prior density of log-rate constants

Let us consider a bimolecular reaction $X_1 + X_2 \to X_3$. For this reaction to occur, two events must take place: one molecule of X_1 must collide with one molecule of X_2 and, given that the two molecules have collided, the reaction must take place. Using basic probabilistic arguments and the well-known hard-sphere collision theory [4], it has been shown in [5] that the probability of a randomly selected pair of molecules X_1 and X_2 at time t to react during an infinitesimal time interval $[t, t + dt)$ is given by cdt. Here, c is known as the specific probability rate constant and is given by

$$c = \frac{\pi (r_1 + r_2)^2}{V} \sqrt{\frac{8 k_B T}{\pi \mu^*}}\, \gamma, \tag{S-1.23}$$

where V is the volume of the biochemical reaction system, T is the temperature, k_B is the Boltzmann constant ($k_B = 1.3806504 \times 10^{-23}$J/K), and γ is the probability that a randomly selected pair of colliding molecules X_1 and X_2 will react. This formula is based on the assumption that each molecule X_n is a hard sphere of radius r_n and mass μ_n. In (S-1.23), $\mu^* = \mu_1 \mu_2 / (\mu_1 + \mu_2)$.

The rate constant k of the previous bimolecular reaction is associated to the specific probability rate constant c by means of $k = AVc$ (for a reaction with different reactants; see [6]), where A is the Avogadro constant ($A = 6.0221415 \times 10^{23}$mol^{-1}). If we assume that a pair X_1 and X_2 of molecules react only after

collision with impact energy greater than E, then $\gamma = \exp(-E/k_B T)$ [5]. In this case,

$$k = \alpha e^{-E/k_B T}, \tag{S-1.24}$$

where

$$\alpha := A\pi(r_1 + r_2)^2 \sqrt{\frac{8k_B T}{\pi\mu^*}}. \tag{S-1.25}$$

Equation (S-1.24) is the well-known Arrhenius formula for the rate constant of a bimolecular reaction, and holds for other types of reactions (e.g., monomolecular and trimolecular) as well. The coefficient α is usually referred to as the pre-exponential factor, or simply the prefactor, whereas, E is referred to as the activation energy of the reaction. In the following, we use (S-1.24) to derive a probabilistic model for the log-rate constants of a biochemical reaction system that leads to an appropriate prior density $p(\boldsymbol{\kappa} \mid \boldsymbol{z})$ for the parameters $\boldsymbol{\kappa}$.

According to (S-1.24), the rate constant of the mth forward reaction is given by

$$k_{2m-1} = \alpha_m e^{-E_m/k_B T}. \tag{S-1.26}$$

Equation (S-1.25) provides a theoretical expression for the prefactor α_m, assuming the ideal situation of both reactant molecules being perfect hard spheres. In reality, however, the situation is much more complex, and we can use (S-1.25) to predict only a portion of the true prefactor value (provided that the masses and radiuses of the reactant molecules are known). As a consequence, we can decompose the prefactor α_m into a predictable part α_m^0 and an unpredictable part ω_m, so that $\alpha_m = \alpha_m^0 \omega_m$. This implies that $\ln \alpha_m = \ln \alpha_m^0 + g_m$, where $g_m := \ln \omega_m$ is a random additive component. The multiplicative factor ω_m can also be used to model unpredictable changes in biochemical conditions or changes in the structure of the reactant molecules, which may also affect the probability of particle collision and thus α_m. Therefore, we will be using the following expression for the prefactor of the mth forward reaction:

$$\alpha_m = \alpha_m^0 e^{g_m}. \tag{S-1.27}$$

We will assume that g_m is a zero-mean Gaussian noise component with standard deviation λ_m. In this case, α_m is a random variable characterized by the log-normal distribution

$$p(\alpha_m) = \frac{1}{\alpha_m \lambda_m \sqrt{2\pi}} \exp\left\{ -\left(\frac{1}{\sqrt{2}\lambda_m} \ln \frac{\alpha_m}{\alpha_m^0} \right)^2 \right\},$$

with parameters $\ln \alpha_m^0$ and λ_m. It has been pointed out in [7] that log-normal distributions are very natural for modeling biochemical processes and are a direct consequence of the thermodynamic behavior of biochemical reaction systems.

Unpredictable changes in biochemical conditions can also affect the probability of reaction after collision, or equivalently, the activation energy E_m. We may consider E_m to be a random variable that is decomposed into two terms: a deterministic term E_m^0 and a random additive term U_m, so that

$$E_m = E_m^0 + U_m, \tag{S-1.28}$$

where $E_m^0, U_m \geq 0$. This is known as the (static) random activation energy (RAE) model [8]. A commonly used probability law for the random energy component U_m is the Maxwell-Boltzmann (exponential) distribution [8]:

$$p(U_m) = \frac{1}{k_B T_m^*} \exp\left\{-\frac{U_m}{k_B T_m^*}\right\}, \quad U_m \geq 0, \tag{S-1.29}$$

at some temperature $T_m^* > T$.[2]

As a consequence of (S-1.26), (S-1.27), and (S-1.28), we have that

$$\kappa_{2m-1} = \kappa_m^0 + g_m - w_m, \quad w_m \geq 0, \tag{S-1.30}$$

where

$$\kappa_m^0 := \ln \alpha_m^0 - \frac{E_m^0}{k_B T} \qquad \text{and} \qquad w_m := \frac{U_m}{k_B T}\,.$$

Moreover, (S-1.29) implies that the probability density function of w_m is given by the following exponential distribution:

$$p(w_m) = \frac{1}{\tau_m}\, e^{-w_m/\tau_m}, \quad w_m \geq 0, \quad \tau_m > 0,$$

where $\tau_m := T_m^*/T > 1$. Note that the expected value of w_m equals its standard deviation, with $\mathrm{E}[w_m] = \mathrm{sd}[w_m] = \tau_m$. In the following, we will assume that, for all $m \in \mathcal{M}$, w_m is statistically independent of g_m. This is a reasonable assumption, considering the fact that these two random variables result from two different biophysical mechanisms, namely molecular collision and molecular reaction, which we may consider to be statistically independent.

[2]It has been suggested in the literature (e.g., in [8]) that T_m^* must be larger than the system temperature T.

The previous modeling steps lead to the following prior probability density function $p(\kappa_{2m-1})$ for the log-rate constant of the mth forward reaction:

$$p(\kappa_{2m-1}) = \frac{e^{\lambda_m^2/2\tau_m^2}}{2\tau_m} \operatorname{erfc}\left[\frac{1}{\sqrt{2}}\left(\frac{\lambda_m}{\tau_m} + \frac{\kappa_{2m-1} - \kappa_m^0}{\lambda_m}\right)\right] e^{(\kappa_{2m-1}-\kappa_m^0)/\tau_m}, \tag{S-1.31}$$

where erfc$[\cdot]$ is the complementary error function [9]. To derive this result, consider a random variable

$$y = c + g - w, \tag{S-1.32}$$

where c is a constant and w, g are two statistically independent random variables so that

$$p_w(w) = \frac{1}{\tau}\, e^{-w/\tau} \text{ (exponential)} \qquad \text{and} \qquad p_g(g) = \frac{1}{\sqrt{2\pi}\lambda}\, e^{-g^2/2\lambda^2} \text{ (Gaussian)}.$$

If we set $u = w - g$, then $y = c - u$ and

$$p_y(y) = p_u(c - y). \tag{S-1.33}$$

Moreover, since w and g are statistically independent, we have [9]

$$\begin{aligned}
p_u(u) &= \int_{-\infty}^{\infty} p_w(x) p_g(x-u)dx \\
&= \int_0^{\infty} \frac{1}{\tau}\, e^{-x/\tau} \frac{1}{\sqrt{2\pi}\lambda} e^{-(x-u)^2/2\lambda^2} dx \\
&= \frac{1}{\sqrt{2\pi}\lambda\tau} \int_0^{\infty} e^{-(\tau x^2 - 2\tau u x + \tau u^2 + 2\lambda^2 x)/2\lambda^2\tau} dx \\
&= \frac{1}{\sqrt{2\pi}\lambda\tau} \int_0^{\infty} e^{-(x+\lambda^2/\tau - u)^2/2\lambda^2} e^{[(\lambda^2/\tau-u)^2 - u^2]/2\lambda^2} dx \\
&= \frac{1}{\tau}\left[\frac{1}{\sqrt{2\pi}\lambda}\int_{\lambda^2/\tau-u}^{\infty} e^{-\xi^2/2\lambda^2} d\xi\right] e^{[(\lambda^2/\tau-u)^2-u^2]/2\lambda^2} \quad \text{(by setting } \xi = x + \lambda^2/\tau - u\text{)} \\
&= \frac{e^{\lambda^2/2\tau^2}}{2\tau} \operatorname{erfc}\left[\frac{1}{\sqrt{2}}\left(\frac{\lambda}{\tau} - \frac{u}{\lambda}\right)\right] e^{-u/\tau}.
\end{aligned} \tag{S-1.34}$$

Finally, by combining (S-1.33) and (S-1.34), we obtain

$$p_y(y) = \frac{e^{\lambda^2/2\tau^2}}{2\tau} \operatorname{erfc}\left[\frac{1}{\sqrt{2}}\left(\frac{\lambda}{\tau} + \frac{y-c}{\lambda}\right)\right] e^{(y-c)/\tau}, \tag{S-1.35}$$

which provides an analytical expression for the probability density function of y. Equation (S-1.31) is now a direct consequence of (S-1.30), (S-1.32), and (S-1.35).

Let us now focus our attention on the log-rate constant κ_{2m} of the mth reverse reaction. Basic thermodynamic arguments imply that

$$k_{2m-1} = k_{2m} \prod_{n \in \mathcal{N}} \phi_n^{s_{nm}}, \tag{S-1.36}$$

where k_{2m-1} and k_{2m} are the rate constants of the mth forward and reverse reactions, respectively, ϕ_n is the capacity of the nth molecular species, and s_{nm} is the stoichiometry coefficients of the nth molecular species associated with the mth reaction. The capacity is a thermodynamic quantity characteristic to a molecular species and depends on the standard chemical potential of that species (see [10] for details). As a consequence, the log-equilibrium constant z_m of the mth reaction depends only on the stoichiometry of the biochemical reaction system and the capacities of the underlying molecular species, since Equation (8) in the Main text and (S-1.36) imply that

$$z_m = \sum_{n \in \mathcal{N}} s_{nm} \ln \phi_n, \quad \text{for all } \; m \in \mathcal{M}.$$

Therefore, z_m is a constant characteristic to the mth reaction.

From Equation (8) in the Main text, note that

$$\kappa_{2m} = \kappa_{2m-1} - z_m, \quad \text{for all } \; m \in \mathcal{M},$$

which implies that κ_{2m} and κ_{2m-1} are two dependent random variables, given z_m. Their joint probability density function satisfies

$$\begin{aligned} p(\kappa_{2m}, \kappa_{2m-1} \mid z_m) &= p(\kappa_{2m} \mid z_m, \kappa_{2m-1}) p(\kappa_{2m-1} \mid z_m) \\ &= \delta(\kappa_{2m} - \kappa_{2m-1} + z_m) p(\kappa_{2m-1}), \end{aligned} \tag{S-1.37}$$

where $\delta(\cdot)$ is the Dirac delta function. Clearly, z_m is a hyperparameter, since it characterizes the prior joint density $p(\kappa_{2m}, \kappa_{2m-1})$ of the log-rate constants of the mth reaction. We will assume here that, given $\boldsymbol{z} = \{z_1, z_2, \ldots, z_M\}$, the reaction rate constants of different reactions are mutually independent. Then, the prior density of the log-rate constants will be given by

$$p(\boldsymbol{\kappa} \mid \boldsymbol{z}) = \prod_{m \in \mathcal{M}} p(\kappa_{2m}, \kappa_{2m-1} \mid z_m) = \prod_{m \in \mathcal{M}} \delta(\kappa_{2m} - \kappa_{2m-1} + z_m) p(\kappa_{2m-1}),$$

by virtue of (S-1.37).

A practical method for determining the hyperparameters $\boldsymbol{\phi}, a, b$

In practice, prior information about the rate constants of a biochemical reaction system may be available from which we may be able to estimate their minimum, maximum, and average values. Moreover, some prior information might be available about the error processes $\eta_n^{(p)}$ in Equation (5) of the Main text, which may allow us to estimate the average value and spread of their variance. In this subsection, we show how to use these values to determine the hyperparameters $\boldsymbol{\phi} = \{\kappa_m^0, \tau_m, \lambda_m, m \in \mathcal{M}\}$ associated with the prior densities $p(\kappa_{2m-1})$ of the forward log-rate constants and the hyperparameters a, b associated with the prior density $p(\sigma^2)$ of the measurement errors.

From (S-1.30), we have that

$$\mathrm{E}[\kappa_{2m-1}] = \kappa_m^0 - \tau_m \qquad \text{and} \qquad \mathrm{sd}[\kappa_{2m-1}] = \sqrt{\lambda_m^2 + \tau_m^2}\,, \tag{S-1.38}$$

by virtue of our assumption that g_m and w_m are statistically independent. Clearly, the parameter κ_m^0 controls the location of $p(\kappa_{2m-1})$, whereas, τ_m controls both location and scale. Moreover, the parameter λ_m controls the scale of $p(\kappa_{2m-1})$, without affecting its location. We illustrate this behavior in Figure S-1.1.

Let κ^{min}, κ^{max} and κ^{avg} be the minimum, maximum, and average values of a forward log-rate constant κ. Our objective is to determine the hyperparameters of the prior density $p(\kappa)$, given by (S-1.31), so that $\mathrm{E}[\kappa] = \kappa^{\text{avg}}$ and $p(\kappa) \simeq 0$, for $\kappa \leq \kappa^{\text{min}}$, $\kappa \geq \kappa^{\text{max}}$.

Since $\mathrm{E}[\kappa] = \kappa^{\text{avg}}$, we must have

$$\kappa^0 - \tau = \kappa^{\text{avg}}, \tag{S-1.39}$$

by virtue of (S-1.38). Moreover, since we want $p(\kappa) \simeq 0$, for $\kappa \leq \kappa^{\text{min}}$, we can impose the condition

$$e^{(\kappa-\kappa^0)/\tau} \leq \tau\epsilon\, e^{-\lambda^2/2}, \quad \text{for } \kappa \leq \kappa^{\text{min}},$$

for a sufficiently small $\epsilon > 0$, which implies that $p(\kappa) < \epsilon$, for $\kappa \leq \kappa^{\text{min}}$, by virtue of (S-1.31) and the facts that $\tau > 1$ and $\mathrm{erfc}(x) \leq 2$. Since $e^{(\kappa-\kappa^0)/\tau}$ is monotonically increasing in κ, it suffices to set $e^{(\kappa^{\text{min}}-\kappa^0)/\tau} = \tau\epsilon^*$, where

$$\epsilon^* := \epsilon\, e^{-\lambda^2/2}, \tag{S-1.40}$$

which, together with (S-1.39), implies that

$$\tau \ln \tau + (1 + \ln \epsilon^*)\tau = \kappa^{\text{min}} - \kappa^{\text{avg}}. \tag{S-1.41}$$

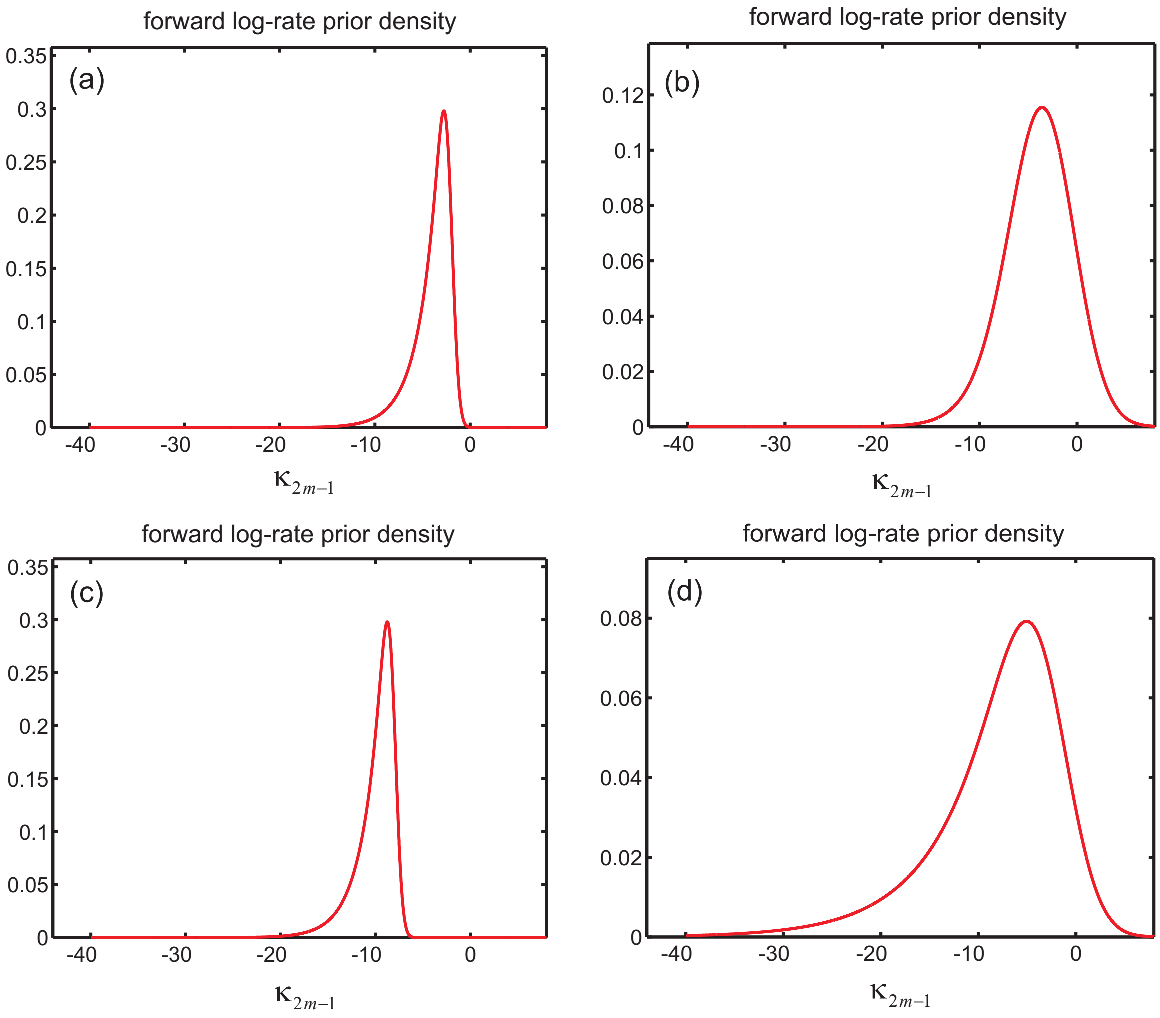

Figure S-1.1: *The prior density $p(\kappa_{2m-1})$ of the forward log-rate constant, given by (S-1.31), when: (a) $\kappa_m^0 = -2$, $\tau_m = 2$, $\lambda_m = 0.6$, (b) $\kappa_m^0 = -2$, $\tau_m = 2$, $\lambda_m = 3$, (c) $\kappa_m^0 = -8$, $\tau_m = 2$, $\lambda_m = 0.6$, and (d) $\kappa_m^0 = -2$, $\tau_m = 6$, $\lambda_m = 3$. Note that κ_m^0 controls the location of $p(\kappa_{2m-1})$, whereas, τ_m controls both location and scale. Moreover, the parameter λ_m controls the scale of $p(\kappa_{2m-1})$, without affecting its location.*

Finally, since we want $p(\kappa) \simeq 0$, for $\kappa \geq \kappa^{\max}$, we can impose the condition

$$\text{erfc}\left[\frac{1}{\sqrt{2}}\left(\frac{\lambda}{\tau} + \frac{\kappa - \kappa^0}{\lambda}\right)\right] \leq 2\tau\epsilon\, e^{-\lambda^2/2}\, e^{-(\kappa-\kappa^0)/\tau}, \quad \text{for } \kappa \geq \kappa^{\max},$$

which implies that $p(\kappa) \leq \epsilon$, for $\kappa \geq \kappa^{\max}$, by virtue of (S-1.31) and the fact that $\tau > 1$. Since $\text{erfc}[(\frac{\lambda}{\tau} + \frac{\kappa-\kappa^0}{\lambda})/\sqrt{2}]$ is monotonically decreasing in κ, it suffices to set

$$\text{erfc}\left[\frac{1}{\sqrt{2}}\left(\frac{\lambda}{\tau} + \frac{\kappa^{\max} - \kappa^0}{\lambda}\right)\right] = 2\tau\epsilon^* e^{-(\kappa^{\max}-\kappa^0)/\tau},$$

which, together with (S-1.39), implies

$$\tau + \lambda\left(r\sqrt{2} - \frac{\lambda}{\tau}\right) = \kappa^{\max} - \kappa^{\text{avg}}, \tag{S-1.42}$$

where

$$r := \text{erfc}^{-1}\left[2\tau\epsilon^{*} e^{-(\kappa^{\max}-\kappa^{\text{avg}}-\tau)/\tau}\right].$$

Given $\kappa^{\min}$, $\kappa^{\max}$, and κ^{avg}, we may be able to determine the values of the hyperparameters κ^{0}, τ, and λ by simultaneously solving (S-1.39), (S-1.41), and (S-1.42). Unfortunately, (S-1.41) and (S-1.42) are nonlinear and they both depend on τ and λ. Hence, finding a solution to these equations is a rather difficult problem [3]. In the following, we discuss a simple approach for determining the values of the hyperparameters, which works quite well.

Equation (S-1.41) depends on λ only through ϵ^{*} [see (S-1.40)]. We can remove this dependence by setting ϵ^{*} to a sufficient small fixed value, such as 0.001. Then, we are left with a nonlinear equation for τ, which we can solve by employing an appropriate numerical method, such as Newton-Raphson [3]. Note that we must choose ϵ^{*} so that the resulting value of τ is greater than one.

Using the value of τ calculated in the previous step, we can calculate the value of λ by solving (S-1.42), in which case

$$\lambda = \frac{\sqrt{2}\, r\tau}{2} \pm \frac{1}{2}\sqrt{2(r^{2}+2)\tau^{2} - 4\tau(\kappa^{\max} - \kappa^{\text{avg}})}\,. \tag{S-1.43}$$

If $\tau < 2(\kappa^{\max} - \kappa^{\text{avg}})/(r^{2}+2)$, we have no real-valued solution for λ. This indicates that we cannot find an appropriate prior density that satisfies the required specifications (i.e., $\text{E}[\kappa] = \kappa^{\text{avg}}$ and $p(\kappa) \simeq 0$, for $\kappa \leq \kappa^{\min}$, $\kappa \geq \kappa^{\max}$). On the other hand, (S-1.43) may produce two different nonnegative values for λ. In this case, we can use both values of λ to evaluate the corresponding prior densities $p(\kappa)$. Then, we can pick the value that leads to a prior density that best satisfies the condition $p(\kappa^{\min}) = p(\kappa^{\max}) = 0$.

After calculating λ by (S-1.43), we must use ϵ^{*} and (S-1.40) to determine the value of ϵ. If the resulting value is sufficiently close to zero, then we can accept the hyperparameter values. Otherwise, we must decrease ϵ^{*} [note from (S-1.40) that $\epsilon \geq \epsilon^{*}$] and repeat the previous procedure. Finally, having computed the values of τ and λ, we can calculate the value of κ^{0} by setting

$$\kappa^{0} = \kappa^{\text{avg}} + \tau. \tag{S-1.44}$$

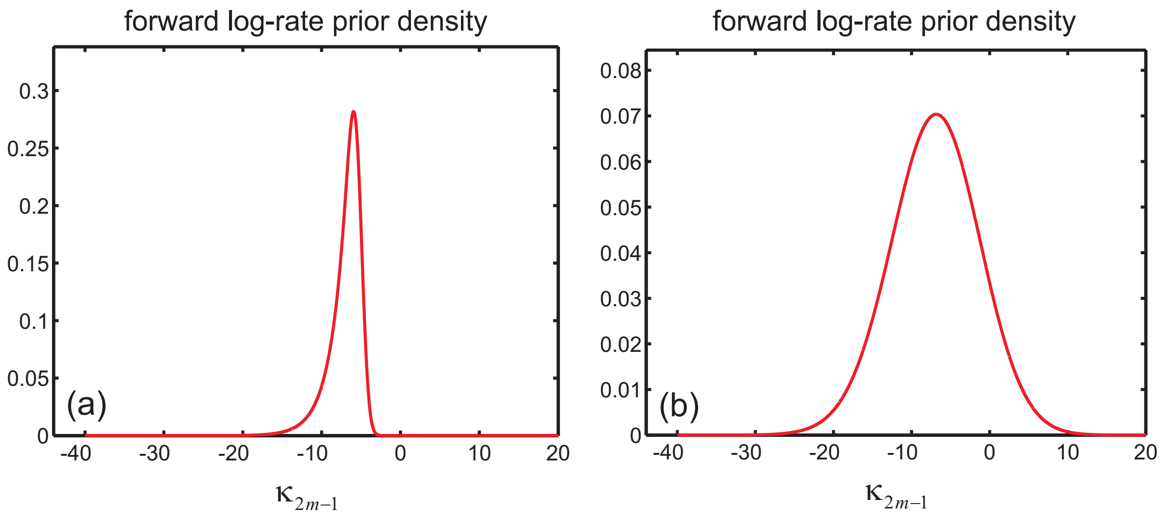

Figure S-1.2: *The prior density $p(\kappa_{2m-1})$ of the forward log-rate constant, given by (S-1.31), when $\kappa_m^0 = -5.1010$, $\tau_m = 1.8990$, and (a) $\lambda_m = 0.7409$, (b) $\lambda_m = 5.3849$. Note that, contrary to our expectations, $p(-17) \not\simeq 0$ and $p(-3) \not\simeq 0$ in (b).*

As an example, let us take $\kappa^{\text{min}} = -17$, $\kappa^{\text{max}} = -3$, and $\kappa^{\text{avg}} = -7$ (these are values we consider in the numerical example), and set $\epsilon^* = 0.001$. Then, (S-1.41) becomes $\tau \ln \tau - 5.9\tau = -10$, which is satisfied with $\tau = 1.8990$. Subsequently, (S-1.43) results in $\lambda = 0.7409$ or $\lambda \simeq 5.3849$, whereas, (S-1.44) gives $\kappa^0 = -5.1010$. The resulting prior density with $\lambda = 0.7409$ is depicted in Figure S-1.2(a). In this case, $\epsilon = 0.0013$, which is sufficiently close to zero. The resulting prior density with $\lambda = 5.3849$ is depicted in Figure S-1.2(b). Clearly, this prior is not acceptable, since it turns out that $p(\kappa^{\text{min}}) = p(-17) \not\simeq 0$ and $p(\kappa^{\text{max}}) = p(-3) \not\simeq 0$.

When the average value, avg, and the spread, sd, of the variance σ^2 of the measurement errors are known, we can uniquely determine the hyperparameters a, b associated with the prior error density $p(\sigma^2)$, given by Equation (9) in the Main text. This is due to the fact that $\text{E}[\sigma^2] = b/(a-1)$ and $\text{var}[\sigma^2] = \{\text{E}[\sigma^2]\}^2/(a-2) = b^2/[(a-1)^2(a-2)]$, for $a > 2$, which imply that

$$a = 2 + \left(\frac{\text{avg}}{\text{sd}}\right)^2$$
$$b = \text{avg} \cdot (a-1).$$

For example, if avg $=$ sd $= 0.5$, then $a = 3$ and $b = 1$, whereas, if avg $= 0.2$ and sd $= 0.1$, then $a = 6$ and $b = 1$. The resulting prior densities are depicted in Figure S-1.3.

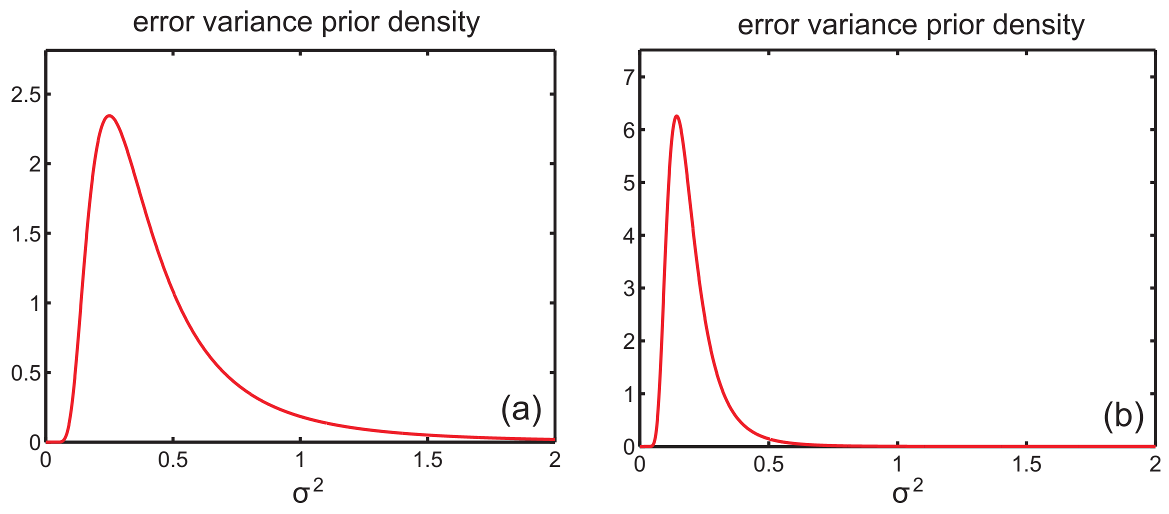

Figure S-1.3: *The prior error variance density* $p(\sigma^2)$*, given by Equation (9) in the Main text, with: (a)* $a = 3$*,* $b = 1$*, and (b)* $a = 6$*,* $b = 1$*. In the first case,* $\mathrm{E}[\sigma^2] = 0.5$ *and* $\mathrm{var}[\sigma^2] = 0.25$*, whereas, in the second case,* $\mathrm{E}[\sigma^2] = 0.2$ *and* $\mathrm{var}[\sigma^2] = 0.01$.

Posterior mode vs. posterior mean

We have mentioned in the Main text that the posterior mode should be a more preferable estimator of the kinetic parameters of a biochemical reaction system than the posterior mean. To see why this is true, suppose that, so long as $|\kappa_{f,m} - \kappa_{f,m}^{\text{true}}| < \epsilon_m$, for $m = 1, 2, ..., M + M_1$, the parameters $\{\boldsymbol{\kappa}_f, \mathbb{W}\boldsymbol{\kappa}_f\}$ reproduce the concentration dynamics of the biochemical reaction system faithfully. Note that a small ϵ_m corresponds to a rate constant that appreciably affects the concentration dynamics, whereas, a large ϵ_m corresponds to a rate constant whose value has little or no effect on the dynamics. If $c(\boldsymbol{\kappa}_f, \boldsymbol{\kappa}_f^{\text{true}})$ is the cost of estimating the true log-rate constants $\boldsymbol{\kappa}_f^{\text{true}}$ by $\boldsymbol{\kappa}_f$, then we can set

$$c(\boldsymbol{\kappa}_f, \boldsymbol{\kappa}_f^{\text{true}}) = \begin{cases} 0, & \text{if } |\kappa_{f,m} - \kappa_{f,m}^{\text{true}}| < \epsilon_m, \quad \text{for } m = 1, 2, ..., M + M_1 \\ 1, & \text{otherwise} . \end{cases} \tag{S-1.45}$$

As a consequence, we would like to find the optimal estimator $\widehat{\boldsymbol{\kappa}}_f$ of $\boldsymbol{\kappa}_f^{\text{true}}$ by minimizing the mean posterior cost $\mathrm{E}[c(\boldsymbol{\kappa}_f, \boldsymbol{\kappa}_f^{\text{true}}) \,|\, \boldsymbol{y}]$ with respect to $\boldsymbol{\kappa}_f$. Note that (S-1.45) implies that

$$\mathrm{E}[c(\boldsymbol{\kappa}_f, \boldsymbol{\kappa}_f^{\text{true}}) \,|\, \boldsymbol{y}] = 1 - \Pr(\{|\kappa_{f,m} - \kappa_{f,m}^{\text{true}}| < \epsilon_m, \text{ for } m = 1, 2, ..., M + M_1\} \,|\, \boldsymbol{y}). \tag{S-1.46}$$

Hence, we can find the optimal estimator $\widehat{\boldsymbol{\kappa}}_f$ by maximizing the probability

$$\Pr(\{|\kappa_{f,m} - \kappa_{f,m}^{\text{true}}| < \epsilon_m, \text{ for } m = 1, 2, ..., M + M_1\} \mid \boldsymbol{y}) = \int_{\kappa_{f,1}-\epsilon_1}^{\kappa_{f,1}+\epsilon_1} \cdots \int_{\kappa_{f,M+M_1}-\epsilon_{M+M_1}}^{\kappa_{M+M_1}+\epsilon_{M+M_1}} p_W(\boldsymbol{\kappa}'|\boldsymbol{y}) d\boldsymbol{\kappa}',$$

with respect to $\boldsymbol{\kappa}_f$.

Clearly, the optimal solution is the center of a hypercube with edge lengths $\{2\epsilon_1, 2\epsilon_2, ..., 2\epsilon_{M+M_1}\}$ with the highest probability given by (S-1.46). When all ϵ_m's are small (i.e., when all parameters appreciably affect the system dynamics), the hypercube will be small as well. In this case, $\widehat{\boldsymbol{\kappa}}_f$ will approximately be the point in the parameter space with the highest posterior probability and, therefore, $\widehat{\boldsymbol{\kappa}}_f = \widehat{\boldsymbol{\kappa}}_f^{\text{mode}}$.

When a parameter does not appreciably affect the system dynamics, the hypercube grows along the corresponding dimension. In this case, the skewness of the posterior density $p_W(\boldsymbol{\kappa}_f \mid \boldsymbol{y})$ may draw the optimal estimator away from the posterior mode along the direction of growth. This however is an acceptable loss of optimality, since the parameter does not appreciably affect the concentration dynamics and finding its optimal value is inconsequential.

References

1. Israel A, Greville TNE: *Generalized Inverses: Theory and Applications*. New York: Springer-Verlag, 2nd edition 2003.
2. Vlad MO, Ross J: **Thermodynamically based constraints for rate coefficients of large biochemical networks**. *WIREs Syst. Biol. Med.* 2009, **1**:348–358.
3. Press WH, Teukolsky SA, Vetterling WT, Flannery BP: *Numerical Recipes: The Art of Scientific Computing*. New York: Cambridge University Press, 3rd edition 2007.
4. Berry RS, Rice SA, Ross J: *Physical Chemistry*. New York: Oxford University Press, 2nd edition 2000.
5. Gillespie DT: **A rigorous derivation of the chemical master equation**. *Physica A* 1992, **188**:404–425.
6. Gillespie DT: **The chemical Langevin equation**. *J. Chem. Phys.* 2000, **113**:297–306.
7. Grönholm T, Annila A: **Natural distribution**. *Math. Biosci.* 2007, **210**:659–667.
8. Vlad MO, Cerofolini G, Oefner P, Ross J: **Random activation energy model and disordered kinetics, from static to dynamic disorder**. *J. Phys. Chem. B* 2005, **109**:21241–21257.
9. Papoulis A, Pillai SU: *Probability, Random Variables and Stochastic Processes*. New York: McGraw Hill, 4th edition 2002.
10. Ederer M, Gilles ED: **Thermodynamically feasible kinetic models of reaction networks**. *Biophys. J.* 2007, **92**:1846–1857.
