# Supplementary material for: Thermodynamically consistent Bayesian analysis of closed biochemical reaction systems
Source: BMC Bioinformatics. 2010 Nov 5;11:547. doi: 10.1186/1471-2105-11-547 (PMC3248051; doi:10.1186/1471-2105-11-547)
Supplement: Additional file 2 — This document contains a detailed description of the computational algorithms used for implementing various steps of the proposed Bayesian analysis approach. [file 1471-2105-11-547-S2.PDF]

ADDITIONAL FILE 2

Thermodynamically consistent Bayesian analysis of closed biochemical reaction systems

COMPUTATIONS

Garrett Jenkinson,[1] Xiaogang Zhong,[2] and John Goutsias*[1]

[1]Whitaker Biomedical Engineering Institute, The Johns Hopkins University, Baltimore, MD 21218, USA
[2]Department of Applied Mathematics and Statistics, The Johns Hopkins University, Baltimore, MD 21218, USA

Email: Garrett Jenkinson - jenkinson@jhu.edu; Xiaogang Zhong - xzhong4@jhu.edu; John Goutsias*- goutsias@jhu.edu;

*Corresponding author

The posterior mode and posterior covariance matrix cannot be calculated analytically. For this reason, we need to develop appropriate computational techniques for their numerical evaluation. It turns out that we can effectively compute the posterior mode by employing an optimization algorithm based on stochastic approximation, and estimate the posterior covariance by sampling the posterior density $p_W(\boldsymbol{\kappa}_f \mid \boldsymbol{y})$, given by Equation (24) in the Main text, using an appropriately designed Monte Carlo method. In this document, we provide a detailed discussion on how to do this.

Computing the prior mode

Evaluating the posterior mode via optimization requires an initial value $\boldsymbol{\kappa}_f(0)$ for the "free" log-rate constants $\boldsymbol{\kappa}_f$. A good choice for such value can be obtained by maximizing the "effective" thermodynamically consistent prior density

$$p_W(\boldsymbol{\kappa}_f, \boldsymbol{\kappa}_d) \propto \delta(\boldsymbol{\kappa}_d - \mathbb{W}\boldsymbol{\kappa}_f) \int p(\boldsymbol{\kappa}_f, \boldsymbol{\kappa}_d \mid \boldsymbol{z}) p(\boldsymbol{z}) d\boldsymbol{z},$$

given by Equation (23) in the Main text. As a consequence of Equation (17) in the Main text and the fact that we replace $p(\boldsymbol{z})$ by the conditional density $p(\boldsymbol{z} \mid \widetilde{\boldsymbol{y}})$, given by Equation (15) in the Main text, we set

$$\begin{aligned}
\boldsymbol{\kappa}(0) &= \arg\max_{\boldsymbol{\kappa}} p_W(\boldsymbol{\kappa}) \\
&= \arg\max_{\boldsymbol{\kappa} \in \mathcal{W}} \int p(\boldsymbol{\kappa} \mid \boldsymbol{z}) p(\boldsymbol{z}) d\boldsymbol{z} \\
&= \arg\max_{\boldsymbol{\kappa} \in \mathcal{W}} \Big[\prod_{m \in \mathcal{M}} p(\kappa_{2m-1}) \Big] \int \Big[\prod_{m \in \mathcal{M}} \delta(\kappa_{2m} - \kappa_{2m-1} + z_m) \Big] p(\boldsymbol{z} \mid \widetilde{\boldsymbol{y}}) d\boldsymbol{z} \\
&= \arg\max_{\boldsymbol{\kappa} \in \mathcal{W}} \Big[\prod_{m \in \mathcal{M}} p(\kappa_{2m-1}) \Big] p(\{z_m = \kappa_{2m-1} - \kappa_{2m}, m \in \mathcal{M}\} \mid \widetilde{\boldsymbol{y}}),
\end{aligned}$$

where $\boldsymbol{\kappa} = \{\boldsymbol{\kappa}_f, \boldsymbol{\kappa}_d\}$ and $\mathcal{W}$ is the thermodynamically consistent region of the parameter space, given by the hyperplane $\boldsymbol{\kappa}_d = \mathbb{W}\boldsymbol{\kappa}_f$. The solution to this problem consists of finding the forward log-rate constants $\{\kappa_{2m-1}(0), m \in \mathcal{M}\}$ that maximize the first term $\prod_{m \in \mathcal{M}} p(\kappa_{2m-1})$, calculating thermodynamically consistent log-equilibrium constants $\{z_m(0), m \in \mathcal{M}\}$ that maximize the second term $p(\boldsymbol{z} \mid \widetilde{\boldsymbol{y}})$, and setting $\kappa_{2m}(0) = \kappa_{2m-1}(0) - z_m(0)$, for $m \in \mathcal{M}$. Note that the Wegscheider conditions, given by Equation (11) in the Main text, are equivalent to the following conditions:

$$z_{m'} = \sum_{m \in \mathcal{M}_1} [\mathbb{S}_{11}^{-1} \mathbb{S}_{12}]_{m,m'} z_m, \quad \text{for every } m' \in \mathcal{M}_2, \tag{S-2.1}$$

by virtue of Equation (8) in the Main text and Equation (S-1.7) in Additional file 1, where $\mathcal{M}_1 = \{1, 2, \ldots, M_1\}$ and $\mathcal{M}_2 = \{M_1 + 1, M_1 + 2, \ldots, M\}$, with $M_1 = \text{rank}(\mathbb{S})$.

To compute the initial forward log-rate constants $\{\kappa_{2m-1}(0), m \in \mathcal{M}\}$, we must find, for each $m \in \mathcal{M}$, the value that maximizes the prior density $p(\kappa_{2m-1})$, given by Equation (16) in the Main text. This problem can be easily solved by a grid search approach that calculates $p(\kappa_{2m-1})$ on a finely spaced uniform grid of points and by detecting the maximum value [1].

To compute a thermodynamically consistent value $\boldsymbol{z}(0)$ that maximizes the conditional density $p(\boldsymbol{z} \mid \widetilde{\boldsymbol{y}})$, given by Equation (15) in the Main text, note that

$$\boldsymbol{z} = \mathbb{G}\boldsymbol{z}^{(1)},$$

by virtue of (S-2.1), where $\boldsymbol{z}^{(1)} = \{z_m, m \in \mathcal{M}_1\}$ and

$$\mathbb{G} = \begin{bmatrix} \mathbb{I}_{M_1} \\ (\mathbb{S}_{11}^{-1}\mathbb{S}_{12})^T \end{bmatrix},$$

with $\mathbb{I}_{M_1}$ being the $M_1 \times M_1$ identity matrix. Maximizing $p(\boldsymbol{z} \mid \widetilde{\boldsymbol{y}})$ with respect to $\boldsymbol{z}$ is now equivalent to maximizing $p(\mathbb{G}\boldsymbol{z}^{(1)} \mid \widetilde{\boldsymbol{y}})$ with respect to $\boldsymbol{z}^{(1)}$. This maximization problem leads to solving the system $\mathbb{U}_0^T\mathbb{G}\boldsymbol{z}^{(1)} = \mathbb{U}_0^T\widetilde{\boldsymbol{y}}$ of linear equations with respect to $\boldsymbol{z}^{(1)}$. A least-squares solution to this problem leads to $\boldsymbol{z}^{(1)}(0) = (\mathbb{U}_0^T\mathbb{G})^\dagger\mathbb{U}_0^T\widetilde{\boldsymbol{y}}$, where $\mathbb{A}^\dagger$ denotes the Moore-Penrose pseudoinverse of matrix $\mathbb{A}$. As a consequence, we have that $\boldsymbol{z}(0) = \mathbb{G}(\mathbb{U}_0^T\mathbb{G})^\dagger\mathbb{U}_0^T\widetilde{\boldsymbol{y}}$.

Finding the posterior mode

It is clear from Equations (21), (22), and (24) in the Main text that, in order to evaluate the mode $\widehat{\boldsymbol{\kappa}}_f^{\text{mode}}$, we need an algorithm for solving the following nonlinear optimization problem:

$$\widehat{\boldsymbol{\kappa}}_f^{\text{mode}} = \arg\max_{\boldsymbol{\kappa}_f} C(\boldsymbol{\kappa}_f \mid \boldsymbol{y}),$$

where $C(\boldsymbol{\kappa}_f \mid \boldsymbol{y}) := D(\boldsymbol{\kappa}_f, \mathbb{W}\boldsymbol{\kappa}_f \mid \boldsymbol{y})$, with

$$\begin{aligned} D(\boldsymbol{\kappa}_f, \boldsymbol{\kappa}_d \mid \boldsymbol{y}) \;=\; & \sum_{m \in \mathcal{M}} \frac{\kappa_{2m-1}}{\tau_m} + \ln\!\left(\operatorname{erfc}\left[\frac{1}{\sqrt{2}}\left(\frac{\lambda_m}{\tau_m} + \frac{\kappa_{2m-1} - \kappa_m^0}{\lambda_m}\right)\right]\right) \\ & -a \ln\left[\frac{2b}{P+1} + \sum_{m \in \mathcal{M}} \sum_{m \in \mathcal{M}'} \theta_{mm'}(\kappa_{2m-1} - \kappa_{2m} - \widetilde{y}_m)(\kappa_{2m'-1} - \kappa_{2m'} - \widetilde{y}_{m'})\right] \\ & - [a + NQ(P+1)/2] \ln\!\left(2b + \sum_{n \in \mathcal{N}} \sum_{q \in \mathcal{Q}} \sum_{p \in \mathcal{P}} [y_n^{(p)}(t_q) - \ln x_n^{(p)}(t_q)]^2\right). \end{aligned} \qquad \text{(S-2.2)}$$

Although a number of different optimization approaches can be employed to solve this problem, we will use here a method based on simultaneous perturbation stochastic approximation (SPSA) [2]. SPSA is a gradient-free ascent algorithm, which estimates the gradient using a finite difference of the objective function evaluated at random perturbations around the current parameter values. The most attractive features of this method are robustness to noise, computational efficiency, and scalability.

The SPSA recursion is given by

$$\boldsymbol{\kappa}_f(i+1) = \boldsymbol{\kappa}_f(i) + \gamma_i \, \boldsymbol{g}_i(\boldsymbol{\kappa}_f(i)), \quad \text{for } \; i = 0, 1, \ldots,$$

where $\{\gamma_i, i = 0, 1, \ldots\}$ is a decreasing sequence of nonnegative numbers and $\{\boldsymbol{g}_i(\boldsymbol{\kappa}_f), i = 0, 1, \ldots\}$ is a sequence of estimators of the gradient of the objective function $C(\boldsymbol{\kappa}_f \mid \boldsymbol{y})$ at point $\boldsymbol{\kappa}_f$. The gradient estimator $\boldsymbol{g}_i$ is a $2M \times 1$ random vector with elements $g_{i,m}$, given by

$$g_{i,m}(\boldsymbol{\kappa}_f) = \frac{C(\boldsymbol{\kappa}_f + \delta_i \boldsymbol{\epsilon}_i \mid \boldsymbol{y}) - C(\boldsymbol{\kappa}_f - \delta_i \boldsymbol{\epsilon}_i \mid \boldsymbol{y})}{2\delta_i \epsilon_{i,m}}, \;\; m = 1, 2, \ldots, 2M, \tag{S-2.3}$$

where $\boldsymbol{\epsilon}_i$ is a $2M \times 1$ random vector with statistically independent random elements $\epsilon_{i,m}$ that follow a ± 1 Bernoulli distribution with equal success and failure probabilities, and $\{\delta_i, i = 0, 1, \ldots\}$ is a decreasing sequence of nonnegative numbers. Parameters γ_i and δ_i should be chosen based on standard guidelines provided in [2]. By following these guidelines, we set

$$\gamma_i = \frac{\gamma}{(i+1+A)^{0.602}} \qquad \text{and} \qquad \delta_i = \frac{\delta}{(i+1)^{0.101}} \, . \tag{S-2.4}$$

We take the value of A to be $1/10$ of the total number of SPSA iterations. Parameter δ can be set at a level that is approximately equal to the standard deviation of the noise in measuring the objective function C. In our case, this standard deviation is directly related to the error tolerance associated with the ODE integrator we use to integrate Equation (2) in the Main text (see also our discussion below). For this reason, we take the value of δ to be the same as the ODE error tolerance. Finally, we choose γ to satisfy the following equation:

$$\frac{\gamma}{(1+A)^{0.602}} \, \mathrm{E}[||\boldsymbol{g}_0(\boldsymbol{\kappa}_f(0))||] = s_0 ||\boldsymbol{\kappa}_f(0)||,$$

where $||\boldsymbol{x}||$ denotes the magnitude of vector $\boldsymbol{x}$. This guarantees that, on the average, the initial SPSA step yields log-rate values within a sufficiently large neighborhood around the initial point $\boldsymbol{\kappa}_f(0)$, measured by the initial search size s_0.

The algorithm for finding the posterior mode proceeds as follows:

Initialization

1. Select values for the hyperparameters $\{\kappa_m^0, \lambda_m, \tau_m, m \in \mathcal{M}\}$, associated with the prior densities $p(\kappa_m)$ of the forward log-rate constants, and values for the hyperparameters $\{a, b\}$, associated with the prior density of the error variance. A practical method for determining these values was discussed in the Additional file 1.

2. Select a desirable number I of SPSA iterations, a desirable level *tol* of ODE error tolerance, and an initial search size s_0 (we set *tol* $= 1 \times 10^{-3}$ and $s_0 = 0.01$).

3. Calculate an initial guess $\boldsymbol{\kappa}_f(0)$ for the "free" log-rate constants by following the approach discussed in the previous section.

4. In (S-2.4), set $A = I/10$, $\delta =$ *tol*, and

$$\gamma = \frac{s_0 ||\boldsymbol{\kappa}_f(0)|| (1 + A)^{0.602}}{\dfrac{1}{L_0} \displaystyle\sum_{l=1}^{L} ||\boldsymbol{g}_0^{(l)}(\boldsymbol{\kappa}_f(0))||},$$

where $\{\boldsymbol{g}_0^{(l)}(\boldsymbol{\kappa}_f(0)), l = 1, 2, \ldots, L_0\}$ are statistically independent realizations of the initial gradient estimator $\boldsymbol{g}_0(\boldsymbol{\kappa}_f(0))$, and L_0 is a sufficiently large integer, so that the denominator in the previous formula provides a sufficiently good approximation of the average initial gradient E$[||\boldsymbol{g}_0(\boldsymbol{\kappa}_f(0))||]$ (we take $L_0 = 10$).

<u>Iteration</u>

For $i = 0, 1, \ldots, I - 1$:

5. Draw $2M$ statistically independent samples $\{\epsilon_{i,m}, m = 1, 2, \ldots, 2M\}$ from a ± 1 Bernoulli distribution with equal success and failure probabilities.

6. By using (S-2.3) and (S-2.4), calculate the $2M$ gradient values $\{g_{i,m}(\boldsymbol{\kappa}_f(i)), m = 1, 2, \ldots, 2M\}$ and use them to calculate new log-rate constant values $\boldsymbol{\kappa}_f(i + 1) = \boldsymbol{\kappa}_f(i) + \gamma_i \boldsymbol{g}_i(\boldsymbol{\kappa}_f(i))$.

Each iteration of the previous optimization algorithm requires computation of the response of the biochemical reaction system under consideration $2(P + 1)$ times [for evaluating the objective function twice]. If parallel computation is available, the system evaluations required by Step 6 can be done independently. If only serial implementation is available, then an effort should be made to reduce the time it takes to integrate the system ODE's.

An important computational trick, which we have implemented with large performance gains, comes from the fact that SPSA enjoys superior performance with noisy objective function evaluations. However, our biochemical reaction system is characterized by deterministic ODE's, which can lead to error-free objective function evaluation, provided that exact integration of these ODE's is possible. We can take advantage of the fact that most ODE integrators have a built-in error tolerance setting that controls the

accuracy of integration. Small error tolerances improve integration accuracy at the expense of increasing computations, whereas, large error tolerances dramatically decrease computations but produce less accurate integrations. Therefore, we can effectively reduce the required computational time by relaxing the ODE error tolerance at the expense of adding "noise" to the evaluation of the objective function. It is expected however that a reasonable amount of "noise" will not appreciably affect the performance of SPSA due to its robustness against inaccurate objective function evaluations [2].

It is a common practice to consider the mode estimator as being the final product $\boldsymbol{\kappa}_f(I)$ of the previous SPSA iterations. However, the value of the objective function C at $\boldsymbol{\kappa}_f(I)$ may not be the largest value obtained during the course of SPSA, due to the fact that SPSA is a stochastic optimization algorithm. An alternative is to consider the mode estimator as being the point in the parameter space at which the value of the objective function becomes maximum during the SPSA iterations, i.e.,

$$\widehat{\boldsymbol{\kappa}}_f^{\text{mode}} = \arg\max \{C(\boldsymbol{\kappa}_f(i) \mid \boldsymbol{y}), i = 0, 1, \ldots, I\} .$$

However, implementation of this equation requires computation of C at each SPSA iteration, which in turn requires an additional number of $I + 1$ system evaluations.

To address this problem, note that evaluation of the gradient $\boldsymbol{g}_i(\boldsymbol{\kappa}_f(i))$, for $i = 0, 1, \ldots, I - 1$, requires computation of the objective function C at points $\boldsymbol{\kappa}_f(i) \pm \delta_i \boldsymbol{\epsilon}_i$, which are proximal to $\boldsymbol{\kappa}_f(i)$. We can therefore approximate the value of the objective function at $\boldsymbol{\kappa}_f(i)$, for $i = 0, 1, \ldots, I - 1$, by averaging the two values $C(\boldsymbol{\kappa}_f(i) \pm \delta_i \boldsymbol{\epsilon}_i \mid \boldsymbol{y})$; i.e., we can set

$$C(\boldsymbol{\kappa}_f(i) \mid \boldsymbol{y}) \simeq \frac{C(\boldsymbol{\kappa}_f(i) + \delta_i \boldsymbol{\epsilon}_i \mid \boldsymbol{y}) + C(\boldsymbol{\kappa}_f(i) - \delta_i \boldsymbol{\epsilon}_i \mid \boldsymbol{y})}{2}, \quad \text{for } i = 0, 1, \ldots, I - 1.$$

Extensive simulations indicate that this modification consistently outperforms the standard SPSA algorithm presented above without requiring additional cost function evaluations.

Estimating the posterior mean and covariance matrix

A potential technique for estimating the posterior mean and covariance matrix is Monte Carlo sampling. This method can be used to estimate posterior expectations of the form $\mathrm{E}[f(\boldsymbol{\kappa}_f) \mid \boldsymbol{y}]$ by generating a large number L of independent and identically distributed (i.i.d.) samples $\{\boldsymbol{\kappa}_f(1), \boldsymbol{\kappa}_f(2), \ldots, \boldsymbol{\kappa}_f(L)\}$, drawn from the posterior distribution $p_W(\boldsymbol{\kappa}_f \mid \boldsymbol{y})$, and by setting

$$\mathrm{E}[f(\boldsymbol{\kappa}_f) \mid \boldsymbol{y}] = \int f(\boldsymbol{\kappa}_f) p_W(\boldsymbol{\kappa}_f \mid \boldsymbol{y}) d\boldsymbol{\kappa}_f \simeq \frac{1}{L} \sum_{l=1}^{L} f(\boldsymbol{\kappa}_f(l)) . \tag{S-2.5}$$

Since the samples are i.i.d., the law of large numbers dictates that an arbitrary degree of estimation accuracy can be achieved by using a sufficiently large number of samples [3].

Unfortunately, this framework is overly restrictive for our problem, since drawing i.i.d. samples from the posterior distribution is a very difficult, if not an impossible, task. An alternative is to use a Markov chain Monte Carlo (MCMC) method, which uses *dependent* samples generated from an ergodic Markov chain converging to $p_W(\boldsymbol{\kappa}_f \mid \boldsymbol{y})$, to estimate the integral in (S-2.5). Indeed, by constructing an appropriate ergodic Markov Chain that generates dependent samples $\{\boldsymbol{\kappa}_f(1), \boldsymbol{\kappa}_f(2), \ldots, \boldsymbol{\kappa}_f(L)\}$, we can guarantee that the sum in (S-2.5) will converge (usually in a mean-square or an almost sure sense) to the posterior mean of $f(\boldsymbol{\kappa}_f)$, as $L \to \infty$ [3].

Although there are several methods for constructing an ergodic MCMC sampling approach, we utilize here the Metropolis algorithm (MA), primarily due to its ease of implementation and known effectiveness in a Bayesian setting. This algorithm proceeds as follows. Given parameters $\boldsymbol{\kappa}_f(l)$ at step l, a new "tentative" set of parameters $\boldsymbol{\kappa}'_f(l)$ is proposed, drawn from a *symmetric* probability distribution $q(\boldsymbol{\kappa}'_f \mid \boldsymbol{\kappa}_f(l))$, satisfying the condition $q(\boldsymbol{\kappa}'_f \mid \boldsymbol{\kappa}_f) = q(\boldsymbol{\kappa}_f \mid \boldsymbol{\kappa}'_f)$, for every $\boldsymbol{\kappa}'_f$ and $\boldsymbol{\kappa}_f$, known as the *proposal* distribution. Then, if $p_W(\boldsymbol{\kappa}'_f(l) \mid \boldsymbol{y}) \geq p_W(\boldsymbol{\kappa}_f(l) \mid \boldsymbol{y})$, we accept $\boldsymbol{\kappa}'_f(l)$ as being the new parameters [i.e., we set $\boldsymbol{\kappa}_f(l+1) = \boldsymbol{\kappa}'_f(l)$]; otherwise, we accept $\boldsymbol{\kappa}'_f(l)$ with probability $p_W(\boldsymbol{\kappa}'_f(l) \mid \boldsymbol{y})/p_W(\boldsymbol{\kappa}_f(l) \mid \boldsymbol{y})$ and reject $\boldsymbol{\kappa}'_f(l)$ [i.e., we set $\boldsymbol{\kappa}_f(l+1) = \boldsymbol{\kappa}_f(l)$] with probability $1 - p_W(\boldsymbol{\kappa}'_f(l) \mid \boldsymbol{y})/p_W(\boldsymbol{\kappa}_f(l) \mid \boldsymbol{y})$.

Note that evaluation of the acceptance/rejection probability requires knowledge of the posterior distribution only up to a constant. This is one reason why MA-MCMC is favorable in a Bayesian setting where it is usually impossible to calculate the proportionality factor associated with the posterior distribution. Another attractive feature is that the proposal distribution can be any symmetric distribution, although choosing this distribution wisely can ensure faster convergence. We can improve the convergence rate if we choose a proposal distribution that results in moderate acceptance rates [2].

Another point worth mentioning here is the *burn-in period* associated with MCMC sampling. The burn-in period is the initial number of MCMC iterations during which the Markov chain has not yet converged to its stationary distribution $p_W(\boldsymbol{\kappa}_f \mid \boldsymbol{y})$. Theoretically speaking, all samples produced by MCMC can be used in (S-2.5). It is however customary to ignore samples during the burn-in period from the computation, hoping that the sum in (S-2.5) will converge faster to the expected value if only samples drawn from the posterior distribution are used.

Unfortunately, it is not easy to accurately determine the burn-in period. Moreover, a large burn-in period may substantially and unnecessarily increase the overall computational effort. Therefore, it would be more attractive if we could initialize the MCMC algorithm with parameters $\boldsymbol{\kappa}_f(1)$ drawn from the posterior distribution $p_W(\boldsymbol{\kappa}_f \mid \boldsymbol{y})$, in which case the burn-in period would be zero, since the Markov chain would be stationary for every $l = 1, 2, \ldots, L$. Of course, this is not possible. However, we can choose $\boldsymbol{\kappa}_f(1)$ to be the posterior mode, in which case we can approximately consider $\boldsymbol{\kappa}_f(1)$ as being a sample drawn from the posterior distribution with the highest probability. This of course will be a good approximation in the ideal case when $\boldsymbol{\kappa}_f(1)$ is indeed the posterior mode and the posterior distribution is tightly clustered around the mode. In practice however the posterior distribution is spread out and we do not know the posterior mode, so $\boldsymbol{\kappa}_f(1)$ is only a local maximum of the posterior distribution found by optimization. Our experience indicates that, by initializing the MCMC sampling algorithm with a local maximum of the posterior distribution obtained by SPSA, we can substantially reduce the number of MCMC iterations required to obtain sufficiently accurate estimates of the posterior mean and covariance matrix.

As a result of the previous discussion, we will adopt the following algorithm for estimating the posterior mean and covariance matrix:

Initialization

1. Select a desirable number L of MA-MCMC iterations.

2. Set $\boldsymbol{\kappa}_f(1) = \widehat{\boldsymbol{\kappa}}_f^{\text{mode}}$, where $\widehat{\boldsymbol{\kappa}}_f^{\text{mode}}$ is obtained after I iterations of the SPSA algorithm discussed in the previous subsection.

3. Set $\xi = 0.1$. Take the proposal distribution $q(\boldsymbol{\kappa}'_f \mid \boldsymbol{\kappa}_f)$ to be the uniform distribution over the hypercube $[\boldsymbol{\kappa}_f - \xi\boldsymbol{e}, \boldsymbol{\kappa}_f + \xi\boldsymbol{e}]$ centered around $\boldsymbol{\kappa}_f$, where $\boldsymbol{e}$ is a vector with all of its elements being equal to one and ξ is a parameter that controls the size of the hypercube in order to achieve a desirable acceptance rate.

Iteration

For $l = 1, 2, \ldots, L$:

4. Draw $2M$ statistically independent samples $\boldsymbol{\epsilon}(l) = \{\epsilon_m(l), m = 1, 2, \ldots, 2M\}$ from the uniform distribution over $[-1, +1]$ and set $\boldsymbol{\kappa}'_f(l) = \boldsymbol{\kappa}_f(l) + \xi\boldsymbol{\epsilon}(l)$.

5. Use (S-2.2) to calculate $C(\boldsymbol{\kappa}'_f(l) \mid \boldsymbol{y})$ and $C(\boldsymbol{\kappa}_f(l) \mid \boldsymbol{y})$ and set $\rho := p_W(\boldsymbol{\kappa}'_f(l) \mid \boldsymbol{y})/p_W(\boldsymbol{\kappa}_f(l) \mid \boldsymbol{y}) = \exp\{C(\boldsymbol{\kappa}'_f(l) \mid \boldsymbol{y}) - C(\boldsymbol{\kappa}_f(l) \mid \boldsymbol{y})\}$.

6. Generate a uniformly distributed random number u over $[0, 1]$.

7. If $\rho \geq u$, set $\boldsymbol{\kappa}_f(l+1) = \boldsymbol{\kappa}'_f(l)$; otherwise, set $\boldsymbol{\kappa}_f(l+1) = \boldsymbol{\kappa}_f(l)$.

Estimation

8. Set

$$\widehat{\boldsymbol{\kappa}}_f^{\text{mean}} = \frac{1}{L} \sum_{l=1}^{L} \boldsymbol{\kappa}_f(l)$$

$$\widehat{\mathbb{V}} = \frac{1}{L} \sum_{l=1}^{L} \left[\boldsymbol{\kappa}_f(l) - \widehat{\boldsymbol{\kappa}}_f^{\text{mode}}\right] \left[\boldsymbol{\kappa}_f(l) - \widehat{\boldsymbol{\kappa}}_f^{\text{mode}}\right]^T.$$

Computing the posterior mode

The objective function $C(\boldsymbol{\kappa}_f \mid \boldsymbol{y})$ is usually not concave, especially when a limited amount of highly noisy data $\boldsymbol{y}$ is available. As a consequence, there is no optimization algorithm that can find the posterior mode in a finite number of steps. However, the following algorithm, which we refer to as maximization-expectation-maximization (MEM) algorithm, performs quite well in our simulations.

Maximization

1. Calculate an initial guess $\boldsymbol{\kappa}_f(0)$ for the log-rate constants by using the previously discussed approach.

2. Perform I SPSA iterations, initialized by $\boldsymbol{\kappa}_f(0)$, to obtain the posterior mode estimate $\widehat{\boldsymbol{\kappa}}_{f,1}^{\text{mode}}$.

Expectation

3. Perform L MA-MCMC iterations, initialized with $\widehat{\boldsymbol{\kappa}}_{f,1}^{\text{mode}}$, to obtain the posterior mean estimate $\widehat{\boldsymbol{\kappa}}_f^{\text{mean}}$.

Maximization

4. Perform I SPSA iterations, initialized by $\widehat{\boldsymbol{\kappa}}_f^{\text{mean}}$, to obtain the posterior mode estimate $\widehat{\boldsymbol{\kappa}}_{f,2}^{\text{mode}}$.

Final Mode Estimate

5. Set $\widehat{\boldsymbol{\kappa}}_f^{\text{mode}} = \arg\max \left\{C(\widehat{\boldsymbol{\kappa}}_{f,1}^{\text{mode}} \mid \boldsymbol{y}), C(\widehat{\boldsymbol{\kappa}}_{f,2}^{\text{mode}} \mid \boldsymbol{y})\right\}$.

A variation of the previous algorithm, which we found to be effective, is to keep track of all objective function evaluations $C(\boldsymbol{\kappa}_f(l) \mid \boldsymbol{y})$, $l = 2, 3, \ldots, L$, during the MA-MCMC (expectation) step, and take

$$\widehat{\boldsymbol{\kappa}}_f^{\text{mode}} = \arg\max \left\{C(\widehat{\boldsymbol{\kappa}}_{f,1}^{\text{mode}} \mid \boldsymbol{y}), C(\boldsymbol{\kappa}_f(l) \mid \boldsymbol{y}), l = 2, 3, \ldots, L, C(\widehat{\boldsymbol{\kappa}}_{f,2}^{\text{mode}} \mid \boldsymbol{y})\right\}.$$

The advantage of this strategy is that it does not waste the objective values evaluated during the MA-MCMC iterations and accounts for the possibility that MA-MCMC may produce parameter values at some iteration that are closer to the actual posterior mode than the parameters obtained by the two SPSA steps.

References

1. Bazaraa MS, Sherali HD, Shetty CM: *Nonlinear programming: Theory and algorithms*. Hoboken, NJ: John Wiley & Sons, 3rd edition 2006.
2. Spall JC: *Introduction to Stochastic Search and Optimization: Estimation, Simulation and Control*. New York: Wiley-Interscience 2003.
3. Liu JS: *Monte Carlo Strategies in Scientific Computing*. New York: Springer-Verlag 2001.
