# Supplementary material for: Thermodynamically consistent Bayesian analysis of closed biochemical reaction systems
Source: BMC Bioinformatics. 2010 Nov 5;11:547. doi: 10.1186/1471-2105-11-547 (PMC3248051; doi:10.1186/1471-2105-11-547)
Supplement: Additional file 3 — In this document, we list the biochemical reactions associated with our numerical example and provide thermodynamically consistent values for the rate constants as well as appropriate values for the initial molecular concentrations. [file 1471-2105-11-547-S3.PDF]

ADDITIONAL FILE 3

Thermodynamically consistent Bayesian analysis of closed biochemical reaction systems

NUMERICAL EXAMPLE

Garrett Jenkinson,[1] Xiaogang Zhong,[2] and John Goutsias*[1]

[1]Whitaker Biomedical Engineering Institute, The Johns Hopkins University, Baltimore, MD 21218, USA
[2]Department of Applied Mathematics and Statistics, The Johns Hopkins University, Baltimore, MD 21218, USA

Email: Garrett Jenkinson - jenkinson@jhu.edu; Xiaogang Zhong - xzhong4@jhu.edu; John Goutsias*- goutsias@jhu.edu;

*Corresponding author

In this document, we list the reactions associated with the biochemical reaction system depicted in Figure 1 of the Main text and provide thermodynamically consistent "true" values for the associated rate constants, as well as appropriate values for the initial concentrations. The example is based on a subset of a well-established model of the EGF/ERK signal transduction pathway proposed by Schoeberl *et al.* [1]. We have obtained published values for the rate constants and initial concentrations from the BioModels database [2].

Model details

The biochemical reaction system depicted in Figure 1 of the Main text is comprised of the following $N = 13$ molecular species:

X_1 : Shc*

X_2 : Grb2

X_3 : Shc*-Grb2

X_4 : Sos

X_5 : Shc*-Grb2-Sos

X_6 : Grb2-Sos

X_7 : (EGF-EGFR*)2-GAP

X_8 : (EGF-EGFR*)2-GAP-Grb2

X_9 : (EGF-EGFR*)2-GAP-Grb2-Sos

X_{10} : Ras-GDP

X_{11} : (EGF-EGFR*)2-GAP-Grb2-Sos-Ras-GDP

X_{12} : Ras-GTP*

X_{13} : (EGF-EGFR*)2-GAP-Grb2-Sos-Ras-GTP ,

which interact by means of the following $M = 9$ reversible association-dissociation reactions:

$$
\begin{aligned}
X_1 + X_2 &\underset{k_2}{\overset{k_1}{\rightleftharpoons}} X_3 \\
X_3 + X_4 &\underset{k_4}{\overset{k_3}{\rightleftharpoons}} X_5 \\
X_2 + X_4 &\underset{k_6}{\overset{k_5}{\rightleftharpoons}} X_6 \\
X_1 + X_6 &\underset{k_8}{\overset{k_7}{\rightleftharpoons}} X_5 \\
X_2 + X_7 &\underset{k_{10}}{\overset{k_9}{\rightleftharpoons}} X_8 \\
X_4 + X_8 &\underset{k_{12}}{\overset{k_{11}}{\rightleftharpoons}} X_9 \\
X_6 + X_7 &\underset{k_{14}}{\overset{k_{13}}{\rightleftharpoons}} X_9 \\
X_9 + X_{10} &\underset{k_{16}}{\overset{k_{15}}{\rightleftharpoons}} X_{11} \\
X_9 + X_{12} &\underset{k_{18}}{\overset{k_{17}}{\rightleftharpoons}} X_{13} \, .
\end{aligned}
$$

Published values for the rate constants can be found in the BioModels database [2]. In particular,

$$\begin{array}{llll} k_1 & = 1.0000 \times 10^{-3} & k_2 & = 33.0000 \\ k_3 & = 3.0000 \times 10^{-3} & k_4 & = 3.8400 \\ k_5 & = 4.5000 \times 10^{-4} & k_6 & = 0.0900 \\ k_7 & = 2.1000 \times 10^{-3} & k_8 & = 12.0000 \\ k_9 & = 1.0000 \times 10^{-3} & k_{10} & = 16.5000 \\ k_{11} & = 1.0000 \times 10^{-3} & k_{12} & = 3.6000 \\ k_{13} & = 4.5000 \times 10^{-4} & k_{14} & = 1.8000 \\ k_{15} & = 1.5000 \times 10^{-3} & k_{16} & = 78.0000 \\ k_{17} & = 2.1000 \times 10^{-4} & k_{18} & = 24.0000 \end{array} \tag{S-3.1}$$

where the forward reaction rates (i.e., the reaction rates with odd subscripts) are measured in cell$/$(molecules $\cdot$ min), whereas, the reverse reaction rates (i.e., the reaction rates with even subscripts) are measured in $1/$min. Unfortunately, these values do not correspond to a thermodynamically feasible biochemical reaction system, since they do not satisfy the Wegscheider conditions, given by Equation (11) in the Main text.

To determine the Wegscheider conditions associated with the previous model [i.e., to determine matrix $\mathbb{W}$ in Equation (S-1.8) of Additional file 1], we must focus on the stoichiometry matrix $\mathbb{S}$, given by

$$\mathbb{S} = \begin{bmatrix} -1 & 0 & 0 & -1 & 0 & 0 & 0 & 0 & 0 \\ -1 & 0 & -1 & 0 & -1 & 0 & 0 & 0 & 0 \\ 1 & -1 & 0 & 0 & 0 & 0 & 0 & 0 & 0 \\ 0 & -1 & -1 & 0 & 0 & -1 & 0 & 0 & 0 \\ 0 & 1 & 0 & 1 & 0 & 0 & 0 & 0 & 0 \\ 0 & 0 & 1 & -1 & 0 & 0 & -1 & 0 & 0 \\ 0 & 0 & 0 & 0 & -1 & 0 & -1 & 0 & 0 \\ 0 & 0 & 0 & 0 & 1 & -1 & 0 & 0 & 0 \\ 0 & 0 & 0 & 0 & 0 & 1 & 1 & -1 & -1 \\ 0 & 0 & 0 & 0 & 0 & 0 & 0 & -1 & 0 \\ 0 & 0 & 0 & 0 & 0 & 0 & 0 & 1 & 0 \\ 0 & 0 & 0 & 0 & 0 & 0 & 0 & 0 & -1 \\ 0 & 0 & 0 & 0 & 0 & 0 & 0 & 0 & 1 \end{bmatrix}.$$

We want to find an $M \times M$ permutation matrix $\mathbb{P}_M$ and an $N \times N$ permutation matrix $\mathbb{P}_N$, such that

$$\mathbb{P}_N \mathbb{S}\, \mathbb{P}_M = \begin{bmatrix} \mathbb{S}_{11} & \mathbb{S}_{12} \\ \mathbb{S}_{21} & \mathbb{S}_{22} \end{bmatrix},$$

where $\mathbb{S}_{11}$ is an $M_1 \times M_1$ invertible matrix, as we have discussed in Additional File 1, with $M_1 = \text{rank}(\mathbb{S})$. Clearly, these permutation matrices are not unique. To find appropriate $\mathbb{P}_M$ and $\mathbb{P}_N$, we first use the reduced row echelon form of the stoichiometry matrix $\mathbb{S}$ and discover that the $M_1 = 7$ columns $\{1, 2, 3, 5, 6, 8, 9\}$

are linearly independent, whereas, the remaining two columns $\{4, 7\}$ linearly dependent on the independent columns of $\mathbb{S}$. Therefore, we are looking for a permutation matrix P_M to rearrange $\mathbb{S}$ so that the first M_1 columns of the resulting matrix are linearly independent. To do so, we must set

$$\mathbb{P}_M = \begin{bmatrix} 1 & 0 & 0 & 0 & 0 & 0 & 0 & 0 & 0 \\ 0 & 1 & 0 & 0 & 0 & 0 & 0 & 0 & 0 \\ 0 & 0 & 1 & 0 & 0 & 0 & 0 & 0 & 0 \\ 0 & 0 & 0 & 0 & 0 & 0 & 0 & 1 & 0 \\ 0 & 0 & 0 & 1 & 0 & 0 & 0 & 0 & 0 \\ 0 & 0 & 0 & 0 & 1 & 0 & 0 & 0 & 0 \\ 0 & 0 & 0 & 0 & 0 & 0 & 0 & 0 & 1 \\ 0 & 0 & 0 & 0 & 0 & 1 & 0 & 0 & 0 \\ 0 & 0 & 0 & 0 & 0 & 0 & 1 & 0 & 0 \end{bmatrix}.$$

By following a similar procedure on the rows of $\mathbb{S}\,\mathbb{P}_M$, we find

$$\mathbb{P}_N = \begin{bmatrix} 1 & 0 & 0 & 0 & 0 & 0 & 0 & 0 & 0 & 0 & 0 & 0 & 0 \\ 0 & 1 & 0 & 0 & 0 & 0 & 0 & 0 & 0 & 0 & 0 & 0 & 0 \\ 0 & 0 & 1 & 0 & 0 & 0 & 0 & 0 & 0 & 0 & 0 & 0 & 0 \\ 0 & 0 & 0 & 1 & 0 & 0 & 0 & 0 & 0 & 0 & 0 & 0 & 0 \\ 0 & 0 & 0 & 0 & 0 & 1 & 0 & 0 & 0 & 0 & 0 & 0 & 0 \\ 0 & 0 & 0 & 0 & 0 & 0 & 0 & 0 & 1 & 0 & 0 & 0 & 0 \\ 0 & 0 & 0 & 0 & 0 & 0 & 0 & 0 & 0 & 1 & 0 & 0 & 0 \\ 0 & 0 & 0 & 0 & 1 & 0 & 0 & 0 & 0 & 0 & 0 & 0 & 0 \\ 0 & 0 & 0 & 0 & 0 & 0 & 1 & 0 & 0 & 0 & 0 & 0 & 0 \\ 0 & 0 & 0 & 0 & 0 & 0 & 0 & 1 & 0 & 0 & 0 & 0 & 0 \\ 0 & 0 & 0 & 0 & 0 & 0 & 0 & 0 & 0 & 0 & 1 & 0 & 0 \\ 0 & 0 & 0 & 0 & 0 & 0 & 0 & 0 & 0 & 0 & 0 & 1 & 0 \\ 0 & 0 & 0 & 0 & 0 & 0 & 0 & 0 & 0 & 0 & 0 & 0 & 1 \end{bmatrix}.$$

As a consequence, we can show that

$$(\mathbb{S}_{11}^{-1}\mathbb{S}_{12})^T = \begin{bmatrix} 1 & 1 & -1 & 0 & 0 & 0 & 0 \\ 0 & 0 & -1 & 1 & 1 & 0 & 0 \end{bmatrix},$$

which, together with Equations (S-1.8) and (S-1.9) in Additional file 1, implies the Wegscheider conditions $\boldsymbol{\kappa}_f = \mathbb{W}\boldsymbol{\kappa}_d$, where the "free" log-rate constants are given by $\boldsymbol{\kappa}_f = \{\kappa_1, \kappa_3, \kappa_5, \kappa_9, \kappa_{11}, \kappa_{15}, \kappa_{17}, \kappa_7, \kappa_{13}, \kappa_2, \kappa_4, \kappa_6, \kappa_{10}, \kappa_{12}, \kappa_{16}, \kappa_{18}\}$, the "dependent" log-rate constants are given by $\boldsymbol{\kappa}_d = \{\kappa_8, \kappa_{14}\}$, and

$$\mathbb{W} = \begin{bmatrix} -1 & -1 & 1 & 0 & 0 & 0 & 0 & 1 & 0 & 1 & 1 & -1 & 0 & 0 & 0 & 0 \\ 0 & 0 & 1 & -1 & -1 & 0 & 0 & 0 & 1 & 0 & 0 & -1 & 1 & 1 & 0 & 0 \end{bmatrix}. \quad \text{(S-3.2)}$$

Since the rate constant values in (S-3.1) are not thermodynamically feasible, we do not use them as the "true" values. Instead, we would like to find thermodynamically feasible parameter values that produce a

dynamic behavior that is similar to the one produced by the published infeasible values. The simplest solution would be to use the published values for $\boldsymbol{\kappa}_f$ and calculate new values for $\boldsymbol{\kappa}_d = \{k_8, k_{14}\}$ by means of $\boldsymbol{\kappa}_d = \mathbb{W}\boldsymbol{\kappa}_f$, with $\mathbb{W}$ given by (S-3.2). Unfortunately, this leads to molecular dynamics that are very different from the dynamics produced by the published system. Since the published values produce dynamics that have been validated on experimental data, we must find a more accurate way for determining thermodynamically feasible rate constant values from a set of infeasible published values.

We can address the previous problem by finding a set of free parameters $\boldsymbol{\kappa}_f$ such that

$$\begin{bmatrix} \mathbb{I}_{M+M_1} \\ \mathbb{W} \end{bmatrix} \boldsymbol{\kappa}_f = \begin{bmatrix} \boldsymbol{\kappa}_f^{\text{pub}} \\ \boldsymbol{\kappa}_d^{\text{pub}} \end{bmatrix},$$

where $\mathbb{I}_{M+M_1}$ is the $(M + M_1) \times (M + M_1)$ identity matrix, whereas, $\boldsymbol{\kappa}_f^{\text{pub}}, \boldsymbol{\kappa}_d^{\text{pub}}$ are the published "free" and "dependent" log-rate constant values, respectively. Unfortunately, no such $\boldsymbol{\kappa}_f$ exists since we know that the published values are thermodynamically infeasible. However, we can calculate the best solution to this problem, in a least-squares sense, given by

$$\boldsymbol{\kappa}_f^{\text{true}} = \begin{bmatrix} \mathbb{I}_{M+M_1} \\ \mathbb{W} \end{bmatrix}^{\dagger} \begin{bmatrix} \boldsymbol{\kappa}_f^{\text{pub}} \\ \boldsymbol{\kappa}_d^{\text{pub}} \end{bmatrix},$$

where $\mathbb{A}^{\dagger}$ is the Moore-Penrose pseudoinverse of matrix $\mathbb{A}$, and compute the remaining "dependent" values by setting $\boldsymbol{\kappa}_d^{\text{true}} = \mathbb{W}\boldsymbol{\kappa}_f^{\text{true}}$. As a result, we obtain the following thermodynamically feasible values for the reaction rate constants:

$$\begin{aligned}
k_1 &= 1.4018 \times 10^{-3} & k_2 &= 23.5420 \\
k_3 &= 4.2053 \times 10^{-3} & k_4 &= 2.7394 \\
k_5 &= 2.0388 \times 10^{-4} & k_6 &= 0.1987 \\
k_7 &= 1.4981 \times 10^{-3} & k_8 &= 16.8210 \\
k_9 &= 1.5746 \times 10^{-3} & k_{10} &= 10.4790 \\
k_{11} &= 1.5746 \times 10^{-3} & k_{12} &= 2.2863 \\
k_{13} &= 2.8579 \times 10^{-4} & k_{14} &= 2.8343 \\
k_{15} &= 1.5000 \times 10^{-3} & k_{16} &= 78.0000 \\
k_{17} &= 2.1000 \times 10^{-4} & k_{18} &= 24.0000
\end{aligned} \qquad \text{(S-3.3)}$$

which we treat as the "true" values.

In Fig. S-3.1, we depict the dynamics of selected molecular species obtained by the published (red curves) and thermodynamically feasible rate values (blue curves). Note that the dynamics do not match perfectly, nor would we expect them to, since the published parameters produce thermodynamically impossible concentration dynamics that a physical system could never produce.

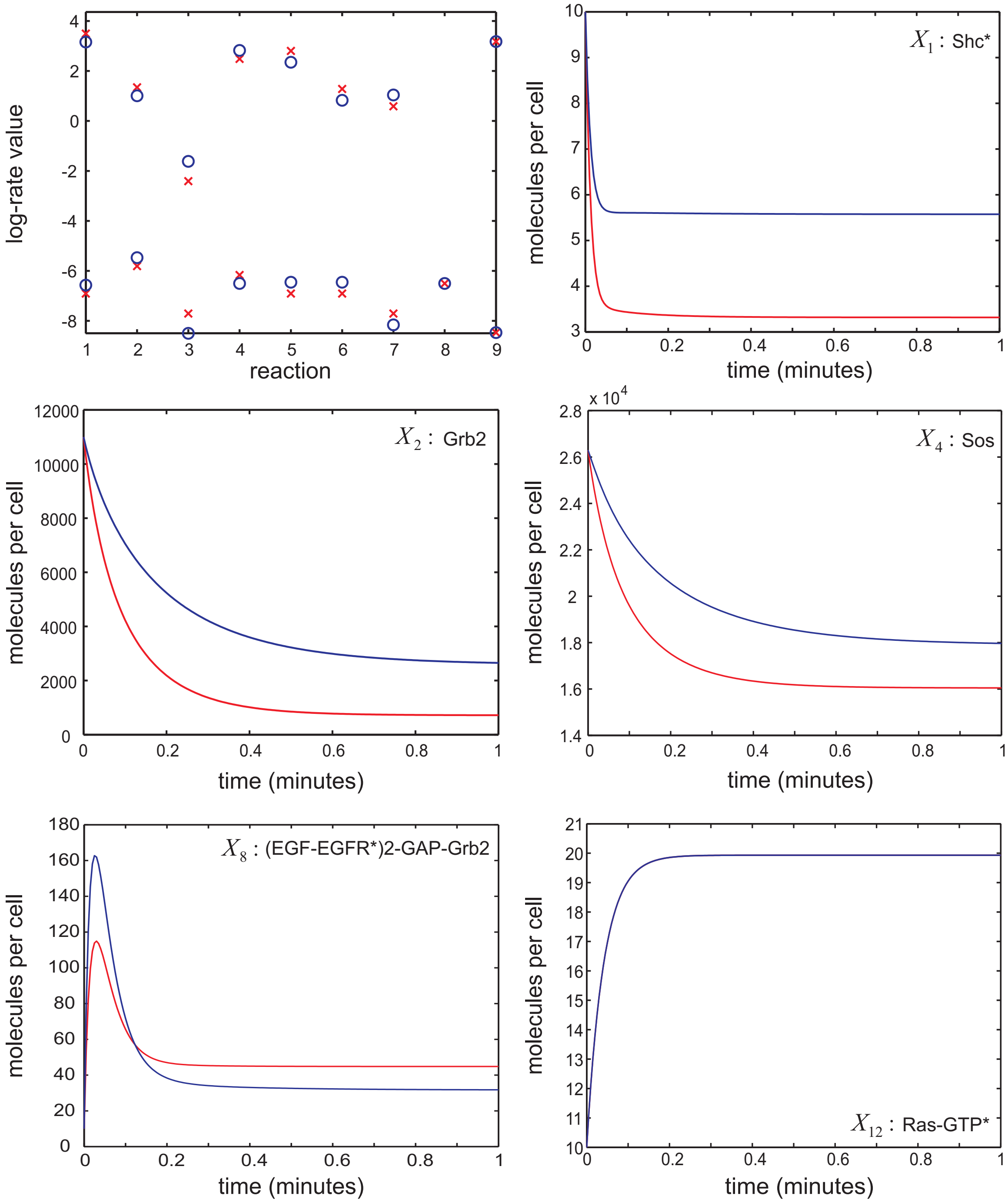

Figure S-3.1: *Published (red) vs. thermodynamically feasible (blue) log-rate values and selected molecular dynamics. Since the published rate values are thermodynamically infeasible, we expect they will result in molecular dynamics that could not be possibly produced by a real biological system. As a consequence, we do not expect perfect match between the "red" and "blue" curves.*

Published values for the initial concentrations of the molecular species can also be found in [2]. Based on these values, we set

$$
\begin{aligned}
c_1 &= 10 \\
c_2 &= 11{,}000 \\
c_3 &= 10 \\
c_4 &= 26{,}300 \\
c_5 &= 10 \\
c_6 &= 40{,}000 \\
c_7 &= 1{,}000 \\
c_8 &= 10 \\
c_9 &= 10 \\
c_{10} &= 72{,}000 \\
c_{11} &= 10 \\
c_{12} &= 10 \\
c_{13} &= 10\,,
\end{aligned}
\tag{S-3.4}
$$

measured in molecules/cell. To compensate for the fact that our biochemical reaction system does not model the entire EGF/ERK signaling cascade, we must account for the upstream EGF stimulus. To do so, we increase the initial concentration of the most upstream molecular species in our model, namely $X_7 =$ (EGF-EGFR*)2-GAP, from 0 in [2] to 1,000 molecules/cell. Finally, we increase the initial concentrations of X_1, X_3, X_5, X_8, X_9, X_{11}, X_{12}, and X_{13} from 0 in [2] to 10, to take into account that, in a real cellular system, these molecular species are constitutively expressed.

References

1. Schoeberl B, Eichler-Jonsson C, Gilles ED, Müller G: **Computational modeling of the dynamics of the MAP kinase cascade activated by surface and internalized EGF receptors**. *Nat. Biotechnol.* 2002, **20**:370–375.
2. Li C, Donizelli M, Rodriguez N, Dharuri H, Endler L, Chelliah V, Li L, He E, Henry A, Stefan MI, Snoep JL, Hucka M, Novère NL, Laibe C: **BioModels database: An enhanced, curated and annotated resource for published quantitative kinetic models**. *BMC Syst. Biol.* 2010, **4**:92.
